# Identification of TRAPPC8 as a Host Factor Required for Human Papillomavirus Cell Entry

**DOI:** 10.1371/journal.pone.0080297

**Published:** 2013-11-14

**Authors:** Yoshiyuki Ishii, Tomomi Nakahara, Michiyo Kataoka, Rika Kusumoto-Matsuo, Seiichiro Mori, Takamasa Takeuchi, Iwao Kukimoto

**Affiliations:** 1 Pathogen Genomics Center, National Institute of Infectious Diseases, Tokyo, Japan; 2 Virology Division, National Cancer Center Research Institute, Tokyo, Japan; 3 Department of Pathology, National Institute of Infectious Diseases, Tokyo, Japan; Centro Nacional de Biotecnologia (CNB-CSIC), Spain

## Abstract

Human papillomavirus (HPV) is a non-enveloped virus composed of a circular DNA genome and two capsid proteins, L1 and L2. Multiple interactions between its capsid proteins and host cellular proteins are required for infectious HPV entry, including cell attachment and internalization, intracellular trafficking and viral genome transfer into the nucleus. Using two variants of HPV type 51, the Ma and Nu strains, we have previously reported that MaL2 is required for efficient pseudovirus (PsV) transduction. However, the cellular factors that confer this L2 dependency have not yet been identified. Here we report that the transport protein particle complex subunit 8 (TRAPPC8) specifically interacts with MaL2. TRAPPC8 knockdown in HeLa cells yielded reduced levels of reporter gene expression when inoculated with HPV51Ma, HPV16, and HPV31 PsVs. TRAPPC8 knockdown in HaCaT cells also showed reduced susceptibility to infection with authentic HPV31 virions, indicating that TRAPPC8 plays a crucial role in native HPV infection. Immunofluorescence microscopy revealed that the central region of TRAPPC8 was exposed on the cell surface and colocalized with inoculated PsVs. The entry of Ma, Nu, and L2-lacking PsVs into cells was equally impaired in TRAPPC8 knockdown HeLa cells, suggesting that TRAPPC8-dependent endocytosis plays an important role in HPV entry that is independent of L2 interaction. Finally, expression of GFP-fused L2 that can also interact with TRAPPC8 induced dispersal of the Golgi stack structure in HeLa cells, a phenotype also observed by TRAPPC8 knockdown. These results suggest that during viral intracellular trafficking, binding of L2 to TRAPPC8 inhibits its function resulting in Golgi destabilization, a process that may assist HPV genome escape from the trans-Golgi network.

## Introduction

Human papillomavirus (HPV) is a non-enveloped virus composed of a circular double-stranded DNA genome of approximately 8000 base pairs (bp) encapsulated by a capsid composed of two structural proteins: the L1 major capsid protein and the L2 minor capsid protein. The capsid is formed from 360 molecules of L1 organized into 72 pentamers, and 12–72 molecules of L2 localized in the central internal cavity of the L1 pentamer [1]. To date, 170 HPV genotypes have been identified in proliferative lesions of the skin or mucosa and classified based on L1 gene sequence homology [2]. HPVs that infect the genital mucosal epithelia are grouped into two types: the low-risk types (*i*.*e*., types 6 and 11) found primarily in condyloma, and the high-risk types (*i.e.*, types 16, 18, 31, 33, 35, 39, 45, 51, 52, 56, 58, 59, 66, 68, and 73) found in cervical cancer, the second most common type of cancer in women worldwide [3].

During infection HPV initially binds to heparan sulfate proteoglycans (HSPGs), either on the cell surface or on the basement membrane, through interactions with L1 [4]–[8]. HPV can also bind to the extracellular matrix (ECM), an interaction involving laminin-332, which may facilitate HPV attachment to the cell [9]. After the initial association of L1 with HSPGs, the capsid undergoes conformational changes, and proteolytic cleavage occurs in L2 [6], [10], [11]. HPV is subsequently transferred to an unidentified cell-surface entry receptor, followed by endocytosis and intracellular trafficking [12]–[20]. In addition, integrin α6β4 [21], [22], CD63 [23], CD151 [24], annexin A2 and S100A10 [25], [26], r-secretase [27], [28], and cyclophilin B [29] play important roles during the entry process. Furthermore, it has been recently proposed that HPV enters the cell through an HSPG-dependent “Trojan horse” mechanism [30]. In this scenario, HPV virions bound to HSPGs on the cell surface are cleaved by matrix metalloprotease, heparinase, or other sheddases, liberating soluble HPV16-HSPG-growth factor complexes into the cell surroundings. These complexes are then incorporated into the cell through cognate growth factor receptor-dependent endocytosis mechanisms involving receptor tyrosine kinase (RTK) and MAP kinase signaling pathways [30].

The endocytotic pathways utilized by HPV for cell entry are dependent on actin dynamics, the Na^+^/H^+^ exchanger and signaling through a variety of kinase pathways, including RTKs, MAP kinase, phosphatidylinositol-3 kinase, protein kinase C, and p21-activated kinases, but are independent of clathrin, caveolin, lipid rafts, or dynamin-2 [19], [31]. After internalization of the virion, the capsid undergoes conformational rearrangements and uncoating in endosomal compartments by acidification [32], and the uncoated L2-DNA complex is delivered to the trans-Golgi network (TGN) by the retromer complex [33], [34]. The L2-DNA complex subsequently travels through an unknown microtubule-dependent route to the nucleus, where replication of the viral genome occurs [11], [17]. L2 is important for the escape of the L2-DNA complex from the endosomal and lysosomal compartments, which is mediated by L2 interaction with sorting nexin 17 [35]–[41]. Although L2 is essential for infectious entry of HPV, precise functional interactions between L2 and host factors are not well defined.

Successful identification of the cellular proteins necessary for a specific phase of a viral life cycle is greatly facilitated by the use of viral variant proteins that are deficient in such processes. L2-dependent infection by HPV type 51 (HPV51) can be investigated using a unique pair of functionally distinct variants: the Ma strain isolated by Matsukura and Sugase, and the Nu strain isolated by Nuovo *et al.* [42]. We have previously reported that pseudovirus (PsV) comprised of the HPV51 MaL1, MaL2 and a reporter plasmid induce efficient reporter expression in inoculated HEK293FT cells, whereas that containing NuL2, instead of MaL2, does not [42], thus suggesting an essential role for MaL2 in HPV infection. Since the Nu strain was originally cloned from a cervical condyloma biopsy [46], it may represent a noninfectious viral genome that was integrated into the cellular genome or maintained only as an episome. In this study, using the two forms of HPV51 L2 as bait, we have performed a proteomic search for cellular proteins responsible for L2-dependent infection and report that the transport protein particle (TRAPP) complex subunit 8, TRAPPC8, coprecipitates with 51MaL2, but not with 51NuL2.

The transport protein particle (TRAPP) complex, also known as a trafficking protein particle complex, is a highly conserved multimeric guanine nucleotide-exchange factor (GEF) that regulates multiple membrane trafficking pathways [43]. Yeast TRAPP complexes exist as three forms: TRAPPI, TRAPPII, and TRAPPIII. TRAPPI and TRAPPII tether coated vesicles during traffic from the endoplasmic reticulum (ER) to the Golgi and during intra-Golgi traffic, respectively. TRAPPIII is required for membrane expansion events during autophagy [44]. TRAPPI is thought to be a core component of the TRAPP complex because both TRAPPII and TRAPPIII contain TRAPPI. In contrast, the mammalian TRAPP complex (mTRAPP) is poorly characterized, and its subunit compositions and functions remain unclear. Among the TRAPP subunits, TRAPPC8 (previously called KIAA1012) is ubiquitously expressed in various human tissues (), and its N-terminal sequence (amino acids [aa] 1–603) is homologous to yeast Trs85, a subunit of the yeast TRAPPIII complex. Several recent studies have revealed that TRAPPC8 is required for autophagy, Golgi stack formation, and ricin susceptibility [45]–[47].

In this paper we demonstrate that TRAPPC8 plays several important roles during HPV infection and that it is an absolute requirement for successful HPV cell entry. We discuss the significance of the interaction between TRAPPC8 and L2 during endocytosis and propose a novel mechanism for its mode of action.

## Materials and Methods

### Cells

HeLa, HaCaT, and HEK293FT cells were cultured in Dulbecco's modified Eagle's medium (DMEM) (Life Technologies Corp. Carlsbad, CA, USA), supplemented with 10% fetal bovine serum (FBS), 100 units/ml penicillin G potassium (Meiji Seika Ltd., Tokyo, Japan), and 60 µg/ml kanamycin sulfate (Wako Pure Chemical Industries Ltd., Tokyo, Japan) at 37°C in 5% CO_2_.

### Plasmids

The plasmids pYSEAP, pEF1α-EGFP, ph51Ma-L1, ph51Ma-L2, ph51Nu-L2, ph51Ch4-L2, ph51Ch5-L2, ph16L1, ph16L2, ph31L1, and ph31L2 were used to construct PsVs as described previously [42], [48], [49]. To generate FLAG-tagged L2 proteins from various strains and chimeras, the L2 coding sequences from HPV51 Ma strain, HPV51 Nu strain, HPV51 chimera 4 (Ch4), HPV51 chimera 5 (Ch5), HPV16 and HPV31 were amplified by polymerase chain reaction (PCR) using the ph51Ma-L2, ph51Nu-L2, ph51Ch4-L2, ph51Ch5-L2, ph16L2, and ph31L2 as templates, respectively. The amplified L2 DNA was inserted into p3xFLAG-CMV™-14 (Sigma-Aldrich, St. Louis, MO, USA) at the *Not* I site. The resultant plasmids were named p3xFLAG14-51MaL2, p3xFLAG14-51NuL2, p3xFLAG14-51Ch4L2, p3xFLAG14-51Ch5L2, p3xFLAG14-16L2, and p3xFLAG14-31L2, respectively. To generate plasmids encoding the L2–green fluorescent protein (GFP) fusion proteins, the GFP coding sequence in the pCMS-EGFP plasmid (Clontech Inc. Mountain View, CA) was amplified by PCR using 5′-CCG CAA GCT TGC GGC CGC GAA TTC ATC GAT AGA TCT GAT ATC GGT ACC AGT CGA CTC TAG AAT GGT GAG CAA GGG CGA GGA GCT G-3′ and 5′-CGG GAA GCT TCC CGG GTT ACT TGT ACA GCT CGT CCA TGC C-3′ as the forward and reverse primers, respectively, and inserted into ph16L2 at the *Hind*III site. L2 DNA from p3xFLAG-51NuL2, -51MaL2, -51Ch4L2, -51Ch5L2, -16L2, or -31L2 was inserted into the GFP plasmid at the *Not*I site. The resultant plasmids were named ph51NuL2-GFP, ph51MaL2-GFP, ph51Ch4L2-GFP, ph51Ch5L2-GFP, ph16L2-GFP and ph31L2-GFP, respectively. To construct plasmids encoding the N-terminal TRAPPC8 (aa 1–603, named N1/603) fused with GST, the TRAPPC8 cording sequence, 1–1809 bp was amplified by PCR using pFIKA1012 (Promega Corp., Madison, WI, USA) as a template DNA. Amplified TRAPPC8 DNA was inserted into pGEX6p-1 (GE Healthcare UK Ltd., Little Chalfont, Buckinghamshire, England) at the *BamH*I and *Sal*I sites. The resultant plasmid was named pGEX6p1-TRAPPC8 N1/603.

### Antigens

To purify N-terminal TRAPPC8 (N1/603) fused with GST (GST-N1/603), *E. coli* BL21 was transformed with pGEX6p1-TRAPPC8 N1/603. The strain was cultured in 1-L LB medium with 1 mM isopropylthio-β-galactoside (IPTG) for 7 h at 16°C and suspended in phosphate-buffered saline (PBS) containing 1 mM phenylmethylsulfonyl fluoride (PMSF). The strain was homogenized with a Bioruptor UCD-200TM (Cosmo Bio Co., Ltd. Tokyo, Japan) in the presence of 1% Trion X-100 and 1 mM DTT. The homogenate was centrifuged at 10,000×*g* for 20 min at 4°C, and the supernatant was applied to a 5-ml GSTrap HP column (GE Healthcare UK Ltd.). The column was washed with PBS containing 1% Triton X-100 and PBS, and then GST-N1/603 bound to the column was eluted with elution buffer (50 mM Tris-HCl pH 8.0, 10 mM glutathione). The eluate was dialyzed against cleaving buffer (50 mM Tris-HCl pH 7.0, 150 mM NaCl, 1 mM EDTA, and 1 mM DTT) and incubated with PreScission™ protease (GE Healthcare UK Ltd.) overnight at 4°C. The cleaved proteins were applied to a GSTrap HP column (GE Healthcare UK Ltd.), and the flow-through fraction containing N1/603 was collected and dialyzed against PBS. TRAPPC8 peptides from aa 880 to 894 (P880/894) and aa 1270 to 1285 (P1270/1285) were synthesized by the Fmoc method (SCRAUM Inc., Tokyo, Japan). P880/894 and P1270/1285 were conjugated with keyhole limpet hemocyanin (KLH) at the cysteine residue of each peptide. HPV51 Ma strain L1 virus-like particles (51MaL1 VLP) were prepared from sf9 cells using the Bac-to-Bac baculovirus expression system (Life Technologies).

### Antibodies

Custom-made rabbit polyclonal antibodies against N1/603, P880/894, and P1270/1285, and custom-made mouse polyclonal antiserum against 51MaL1 VLP were obtained from SCRUM Inc.

### Immunoprecipitation

HEK293FT cells, which had been seeded in a 10-cm culture dish 16 h before transfection, were transfected with 20-μg p3xFLAG14-51MaL2, p3xFLAG14-51NuL2, p3xFLAG14-51Ch4L2, p3xFLAG14-51Ch5L2 p3xFLAG14-16L2, or p3xFLAG14-31L2 using 80-μl Fugene HD (Roche, Diagnostics GmbH, Mannheim, Germany). Two days later, the cells were washed with PBS and harvested using a cell scraper. The cells were then suspended in 2-ml IP buffer (20 mM Tris-HCl pH 8.0, 10% glycerol, 5 mM MgCl_2_, 0.1% Tween 20, 0.1 M KCl, 0.5 mM DTT and protease inhibitor cocktail; Complete Mini, Roche Diagnostics GmbH), and briefly sonicated with a UP 50H ultrasonic processor (Hielscher Ultrasonics GmbH, Teltow, Germany). The homogenized cells were centrifuged at 5000×*g* for 10 min at 4°C. The supernatant was mixed with 100-μl anti-FLAG M2 agarose beads (A2220, Sigma-Aldrich) and rotated for 1 h at room temperature. Proteins bound to the beads were eluted with 100-μl IP buffer containing 250 µg/ml 3xFLAG peptides (Sigma-Aldrich).

### Identification of proteins precipitated with 51MaL2, but not 51NuL2

The proteins released from the anti-FLAG M2 agarose beads were boiled in 6× SDS sample buffer for 10 min and fractionated using 5–20% gradient SDS-polyacrylamide gel electrophoresis (PAGE) (ATTO, Tokyo, Japan), followed by SYPRO Ruby (Life Technologies) staining. The 150-kDa protein band that co-precipitated with 51MaL2 was excised from the gel and subjected to in-gel trypsin digestion. The resultant peptide mixtures were analyzed by MALDI-QIT-TOF MS (AXIMA-QIT, Shimazu Biotech, Japan). Mascot software (Matrix Science) was used for protein identification.

### Western blot assay

The lysate proteins were separated by SDS-PAGE and transferred to a polyvinylidene difluoride (PVDF) membrane using an IBlot™ gel transfer system (Life Technologies). TRAPPC8 was detected using rabbit polyclonal anti-N1/603, anti-P880/894, or anti-P1270/1285 antibodies at a 1∶200 dilution, followed by anti-rabbit IgG-HRP (Santa Cruz Biotechnology Inc., Santa Cruz, CA, USA). TRAPPC12 was detected using mouse polyclonal TRAPPC12 antibody (Anti-TTC15, ab88751, Abcam, Cambridge, UK), followed by anti-mouse IgG-HRP (Santa Cruz Biotechnology Inc.). Alpha-tubulin was detected using mouse monoclonal antibody (T-9026, Sigma-Aldrich Co., St. Louis, MO, USA), followed by anti-mouse IgG-HRP (Santa Cruz Biotechnology Inc.). Immunoreactive bands were visualized using an ECL Plus Western blot detection system (GE Healthcare UK Ltd.) and a Typhoon 9410 imager (GE Healthcare UK Ltd.).

### TRAPPC8- or TRAPPC12-knockdown cells

HeLa (2.5×10^4^) or HaCaT (5×10^4^) cells were transfected with 12.5 pmol siGENOME non-targeting siRNA (D-001210-01-05, Thermo Fisher Scientific Inc., MA, USA); siGENOME set of 4, human TRAPPC8 (KIAA1012, MQ-010645-00-0002, Thermo Fisher Scientific) or siGENOME set of 4 human TRAPPC12 (TTC15, MQ-016861-00-0002; Thermo Fisher Scientific) using 1 µl Dharma FECT 1 transfection reagent (Thermo Fisher Scientific) in a 24-well plate. Two or 3 days post-transfection, the cells were inoculated with PsVs or HPV31 authentic virions.

### Production of PsVs and reporter assay

51PsVNuL2, 51PsVMaL2, 16PsV, or 31PsV, composed of HPV51 MaL1/MaL2, HPV51 MaL1/NuL2, HPV16 L1/L2, or HPV31 L1/L2, and a reporter plasmid encoding enhanced green fluorescence protein (pEF1α-EGFP), were produced as described previously [42], [48], [49]. HeLa cells (1×10^5^/well) in a 24-well plate were inoculated with the PsV stock diluted with growth medium to 1∶5000 (MOI of ∼50 particles/cell). After incubation for 2 days, the cells were harvested with trypsin treatment, and GFP-positive cells were counted by flow cytometry (PERFLOW; Furukawa Electric Co. Ltd., Tokyo, Japan). To determine the viability of HeLa cells, WST-1 (Roche, Diagnostics GmbH) was added to the culture medium at 10-fold dilution. After incubation in 5% CO_2_ for 30 min at 37°C, the A_450_ values of media were measured with a microplate reader (Labsystems iEMS Reader MF, Thermo Electron Corporation, Helsinki, Finland).

### Production of HPV31b virions and infection assay

HPV31b virions were prepared from CIN612 9E raft tissues [50]. The raft tissues grown for 14 days at the air–liquid interface were suspended in suspension buffer (D-PBS containing 0.85 M NaCl), and stored at −80°C. Frozen tissues were homogenized with a Mixer Mill MM300 (Qiagen, Hilden, Germany) at 25.0/s frequency twice for 5 min each. Homogenates were centrifuged at 8,000×*g* for 10 min at 4°C, and the supernatants were collected. Pellets were resuspended in suspension buffer and homogenized as above. Homogenates were centrifuged and the supernatants were collected. These steps were repeated three times. The pooled supernatant was placed on an Optiprep (Axis-Shield PoC AS, Oslo, Norway) gradient (from top to bottom, 27%, 33%, and 39% in PBS containing 1 mM CaCl_2_, 10 mM MgCl_2_, and 0.85 M NaCl) and centrifuged at 237,000×*g* for 3.5 h at 16°C in an SW55Ti rotor (Beckman Coulter, Fullerton, CA, USA). Fractions (400 µl each) were obtained by puncturing the bottom of the tube. Aliquots (5 µl per fraction) were analyzed by Western blot using mouse anti-HPV16L1 antibody (554171, BD Pharmingen, San Diego, CA, USA), which can also recognizes HPV31 L1. The fraction in which L1 was most abundant was used as a stock of HPV31b virions.

HaCaT cells (2×10^5^/well) in a 24-well plate were inoculated with HPV31b virions diluted with growth medium to 1∶100. The medium was changed daily. After incubation for 3 days, total RNA was extracted from the cells using an RNeasy mini prep kit (Qiagen). Reverse transcription (RT) and quantitative PCR (qPCR) were performed using a TaqMan Gene-Expression Cells-to-Ct™ kit (Life Technologies) with HPV31E1ˆE4 primers (E7.4A and E4.3B) [51] (final concentration 200 nM) and HPV31 E1ˆE4 probe (6FAM-CAG TGA CGA AAT ATC CTT TGC TGG GAT TGT T-TAMRA) [52] (final concentration 100 nM) on a 7900HT fast real-time PCR system (Life Technologies). The qPCR cycling profile was as follows: 50°C for 2 min, 95°C for 10 min, 45 cycles at 95°C for 15 s, and 60°C for 1 min. The standard curve was constructed using serial 10-fold dilutions of an E1ˆE4 cDNA template from a cloned sequence. Relative HPV31E1ˆE4 expression was normalized to beta-actin mRNA using TaqMan beta-actin control reagents (401846, Life Technologies).

### Flow cytometric analysis

HeLa cells post-transfected with siRNAs in a 24-well plate were detached with PBS containing 2.5 mM EDTA. The cells (2×10^5^) were suspended in 100-μl PBS containing 10% FBS and 5-μl anti-P880/894, anti-P1250/1270, or anti-N1/603 antibodies. Cells were then incubated at 4°C for 1 h with tapping every 10 min. After washing with PBS containing 10% FBS, the cells were suspended in 100-μl PBS containing 10% FBS and 0.1-μl anti-rabbit IgG conjugated to Alexa Fluor 488 (Life Technologies). The cells were then incubated at 4°C for 1 h, with tapping every 10 min. After washing with PBS containing 10% FBS, the fluorescence was measured with a flow cytometer (PERFLOW; Furukawa Electric Co. Ltd., Tokyo, Japan).

### Immunofluorescence microscopy

For observation of cell-surface TRAPPC8, HeLa cells (4×10^4^) post-transfected with siRNAs in an 8-well chamber glass slide were inoculated with 51PsVs (MOI of ∼2000 particles/cell) in 200-μl growth medium. The cells were then incubated at 4°C for 1 h and washed with the medium. Cells were incubated in the medium with mouse anti-51L1 antiserum and rabbit anti-P880/894 antibody at 4°C for 1 h, followed by staining with Alexa Fluor 488-conjugated goat anti-mouse IgG and Alexa Fluor 546-conjugated goat anti-rabbit IgG (Life Technologies) at 4°C for 1 h. The cells were washed with the medium and fixed with 4% paraformaldehyde (PFA) in PBS for 15 min at room temperature. After washing with PBS, the cells were mounted using a Prolong Gold anti-fade reagent with DAPI (Life Technologies).

For investigation of subcellular localization of L1, packaged DNA, and TGN46 in cells inoculated with 51PsVs, HEK293FT cells in an 8-well chamber glass slide were inoculated with PsVs (MOI of ∼2000 particles/cell) containing 5-ettynil-2′-deethynil-2′-deoxyuridine (EdU)-labeled DNA in 200 µl growth medium [48]. The cells were incubated at 4°C for 1 h and washed with the medium, incubated in the medium at 37°C, fixed with 4% PFA in PBS for 15 min at room temperature and permeabilized with 0.5% Triton X-100 in PBS for 20 min at room temperature. The cells were washed with 3% BSA in PBS and incubated with a Click-it reaction cocktail containing Alexa Fluor 488 (Click-it™ EdU imaging kit; Life Technologies) as described previously [48]. The cells were incubated with mouse anti-51L1 antiserum and rabbit anti-TGN46 antibody (ab50595) (Abcam, Cambridge, UK), followed by staining with Alexa Fluor 555-conjugated goat anti-mouse IgG (Life Technologies) and Alexa Fluor 647-conjugated goat anti-rabbit IgG (Life Technologies), and washed and mounted as described above. To visualize the Golgi, the cells were incubated with mouse anti-GM130 antibody (610822, BD Biosciences), followed by staining with Alexa Fluor 555-conjugated goat anti-mouse IgG (Life Technologies).

To assess the effect of L2 on Golgi stacks in cells, HeLa cells were transfected with ph51NuL2-GFP, ph51MaL2-GFP, ph51Ch4L2-GFP, ph51Ch5L2-GFP, ph16L2-GFP or ph31L2-GFP using Lipofectamine 2000 (Life Technologies) and incubated at 37°C for 24 h. These cells were fixed with 4% PFA in PBS for 15 min at room temperature and permeabilized with 0.5% Triton X-100 in PBS for 20 min at room temperature. The cells were washed with 3% BSA in PBS and incubated with mouse anti-GM130 antibody (610822, BD Biosciences), followed by staining with Alexa Fluor 555-conjugated goat anti-mouse IgG (Life Technologies), and washed and mounted as described above.

All fluorescent images were obtained using a FluoView FV1000 confocal microscope (Olympus, Tokyo, Japan).

### Cell attachment and internalization assay with PsVs

HeLa cells at 72 h post-transfection with control or TRAPPC8 (KIAA1012-04) siRNA were inoculated in a 24-well plate with 1 µg of 51PsVMaL2, 51PsVNuL2, 51PsVL2–, 16PsV, 16PsVL2–, 31PsV or 31PsVL2– in 200 µl growth medium (MOI of ∼2000 particles/cell). After incubation at 4°C for 1 h, the cells were washed and incubated in 500-μl medium at 37°C for 0, 1, 2, 4, or 8 h. At each time point, the cells were detached with PBS containing 2.5 mM EDTA (Trypsin –) or 0.2% Trypsin with 1 mM EDTA (Trypsin +). Detached cells were lysed and boiled. HPV51 L1, HPV16/31 L1, TRAPPC8, and α-tubulin in the cell lysates were detected by Western blotting using the anti-51L1 antiserum, anti-HPV16L1 (554171, BD Biosciences), anti-TRAPPC8 (anti-N1/603), and anti-α-tubulin antibodies, respectively.

### Internalization assay with transferrin and cholera toxin

HeLa cells at 72 h post-transfection with control or TRAPPC8 (KIAA1012-04) siRNA were incubated with 20 µg/ml transferrin (Tf) conjugated with Alexa Fluor 568 (T23365, Life Technologies) at 4°C for 1 h then followed by incubation at 37°C for 15 min, or incubated with 1 µg/ml cholera toxin subunit B (CtxB) conjugated with Alexa Fluor 555 (C34776, Life Technologies) at 4°C for 1 h then followed by incubation at 37°C for 1 h. Endocytotic uptake of the ligands was terminated by washing the cells with ice-cold PBS, and surface-bound Tf and CtxB were removed by acid treatment with DMEM containing 25 mM sodium acetate (pH 2.0) on ice for 2 min [53]. The cells were fixed, permeabilized, and incubated with mouse anti-GM130 antibody (610822, BD Biosciences) and anti-mouse IgG conjugated with Alexa Fluor 488 (Life Technologies). Fluorescence derived from incorporated Tf and CtxB was measured by confocal microscopy and quantified with FV1000 software (Olympus, Tokyo, Japan).

## Results

### Identification of TRAPPC8 as an L2-interacting protein

**Figure 1:**
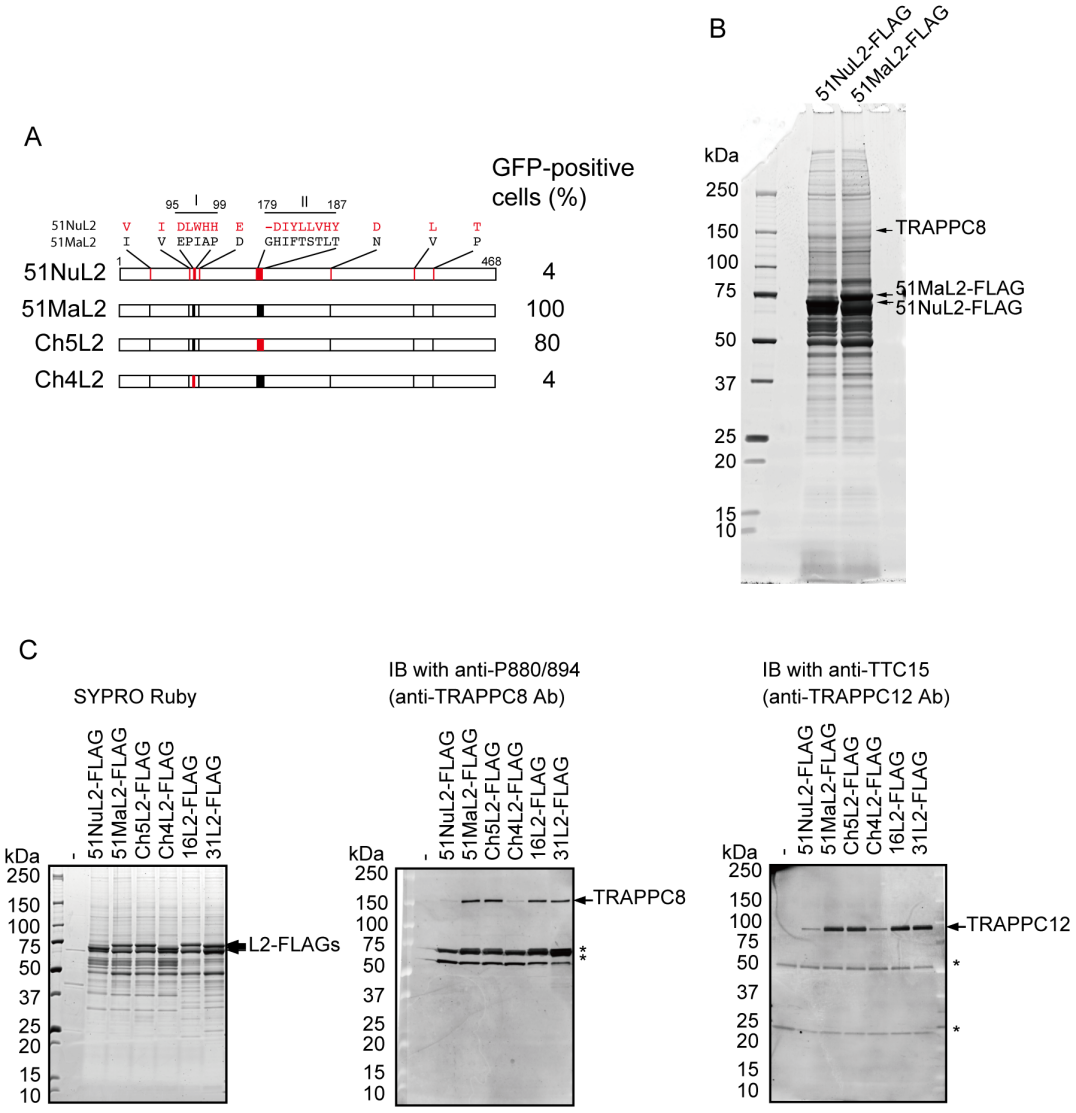
TRAPPC8 coprecipitation with L2. (A) Schematic diagram of HPV51 L2s. The percentage of cells expressing GFP after inoculation with PsVs containing GFP-expression plasmid, HPV51 Ma L1, and each L2 are shown on the right. (B) Electrophoresis of subcellular proteins that coprecipitated with FLAG-tagged 51NuL2 (51NuL2-FLAG) or FLAG-tagged 51MaL2 (51MaL2-FLAG). Proteins were stained with SYPRO Ruby. (C) TRAPPC8 and TRAPPC12 coprecipitated with FLAG-tagged HPV51 L2s (51NuL2-FLAG, 51MaL2-FLAG, Ch5L2-FLAG, Ch4L2-FLAG), HPV16 L2 (16L2-FLAG), and HPV31 L2 (31L2-FLAG). Precipitated L2s were stained with SYPRO Ruby (left panel). Subcellular proteins coprecipitated with L2s were analyzed by Western blotting using anti-TRAPPC8 antibody (Anti-P880/894) (middle panel) or anti-TRAPPC12 antibody (Anti-TTC15) (right panel). Asterisks: unknown proteins cross reactive with the antibodies.

We used HPV51 L2s derived from strains Ma and Nu as bait in the identification of the cellular proteins mediating L2-dependent HPV infection. HEK293FT cells were transfected with an expression plasmid for FLAG-tagged NuL2 (51NuL2-FLAG) or MaL2 (51MaL2-FLAG). Two days later the cells were homogenized to yield lysates. 51NuL2-FLAG or 51MaL2-FLAG were immunoprecipitated with anti-FLAG-antibody, followed by protein separation by SDS-PAGE. A comparison of the resolved proteins on a SYPRO Ruby-stained gel revealed a 150-kDa protein band present only in the 51MaL2-FLAG precipitate (Figure 1B). Peptide mass fingerprint analysis identified this protein as TRAPPC8. Western blotting analysis confirmed this identification; an anti-TRAPPC8 antibody (anti-P880/894), which targeted a TRAPPC8 peptide corresponding to aa 880–894 (P880/894), recognized a 150-kDa protein in the 51MaL2-FLAG precipitate, but not in the 51NuL2-FLAG precipitate (Figures 1C, middle panel).

The aa sequence of MaL2 (GenBank GQ487712) is strikingly different from that of NuL2 (GenBank M62877) in two regions: aa 95–99 (region I) and aa 179–187 (region II) (Figure 1A). Using HPV51 PsVs containing a series of chimeric Ma and Nu L2s, we have previously reported that region I of MaL2, which shows a high degree of identity among mucosal HPVs, is indispensable for PsV-mediated gene transduction [42]. Western blotting analysis of proteins co-immunoprecipitated with FLAG-tagged chimeric L2s, Ch5L2 (Ch5L2-FLAG), in which region II of MaL2 was replaced by that of NuL2, and Ch4L2 (Ch4L2-FLAG), in which region I of MaL2 was replaced by that of NuL2, showed the presence of TRAPPC8 in the Ch5L2-FLAG precipitate but not in the Ch4L2-FLAG precipitate (Figures 1C, middle panel). Flow cytometric analyses of HeLa cells inoculated with PsVs containing these chimeric L2s and a GFP-expression plasmid revealed that 80% of HeLa cells inoculated with PsV containing the Ch5L2 were GFP-positive, whereas only 4% were GFP-positive in cells inoculated with PsV containing the Ch4L2 (Figure 1A). These results indicate that the transgene expression levels generated by PsVs containing chimeric L2s correlate with the protein level of TRAPPC8 that coprecipitated with L2, suggesting that the TRAPPC8 interaction with MaL2 through region I is essential for transgene expression.

Furthermore, TRAPPC8 co-immunoprecipitated with FLAG-tagged L2s of HPV16 and HPV31 at levels similar to those observed with FLAG-tagged MaL2 (Figures 1C, middle panel), suggesting that the interaction between L2 and TRAPPC8 is a general property of L2 in various HPV types.

Since TRAPPC8 is supposed to be a subunit of the TRAPPIII complex, we further examined coprecipitation of TRAPPC12, another subunit of TRAPPIII subunits, with L2s. TRAPPC12 coprecipitated with FLAG-tagged L2s able to bind to TRAPPC8 (Figure 1C, right panel).

### Requirement of TRAPPC8 for the early stages of HPV infection

**Figure 2:**
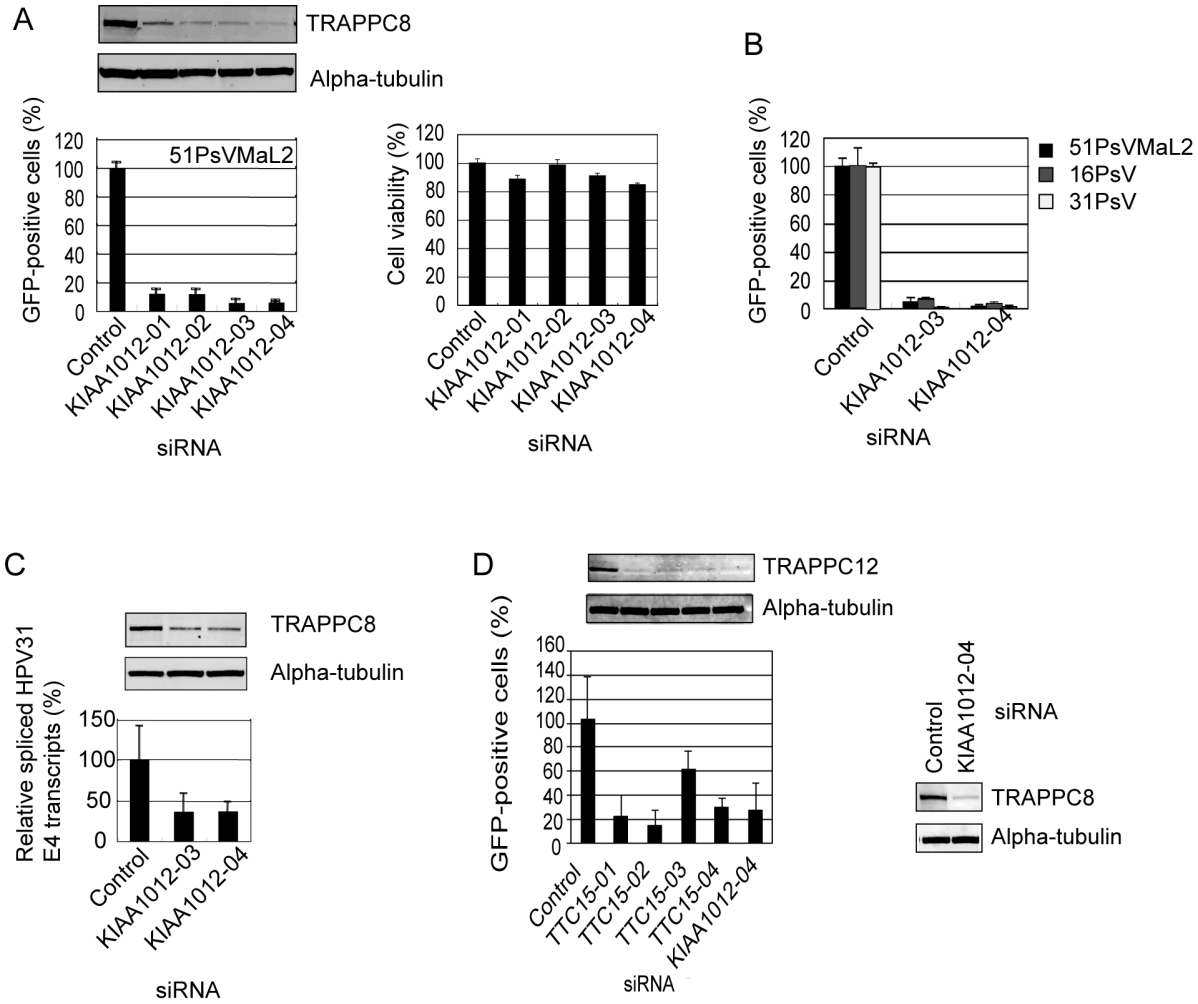
Effects of TRAPPC8 knockdown on gene transduction of PsVs and infection with authentic HPV31 virions. (A) HeLa cells were transfected with individual TRAPPC8 siRNAs (KIAA1012-01, -02, -03, or -04). At 2 days after siRNA transfection the cells were inoculated with 51PsVMaL2 (MOI of ∼50 particles/cell). Following incubation for 2 days, the number of cells expressing GFP was measured by flow cytometry (lower left panel). Top panels: Western blotting of cells transfected with the indicated TRAPPC8 siRNAs using anti-TRAPPC8 antibody (Anti-N1/603) and anti-α-tubulin antibodies as a loading control. Lower right panel: viability of HeLa cells transfected with the indicated TRAPPC8 siRNAs was analyzed using a WST-1 cell proliferation assay at 96 h post-transfection. (B) HeLa cells were transfected with the indicated TRAPPC8 siRNAs (KIAA1012-03, or -04). At two days after siRNA transfection the cells were inoculated with 51PsVMaL2, 16PsV or 31PsV (MOI of ∼50 particles/cell). Following incubation for 2 days, the number of cells expressing GFP was measured by flow cytometry. (C) HaCaT cells were transfected with the indicated TRAPPC8 siRNAs (KIAA1012-03 or -04). At two days after siRNA transfection the cells were inoculated with HPV31b virions prepared from CIN612-9E raft tissues. Following incubation for 3 days, E1ˆE4 viral transcript was quantified by RT-qPCR. The level of the HPV31E1ˆE4 transcript was normalized to that of β-actin mRNA. E1ˆE4 transcript relative to beta-actin mRNA in control siRNA transfected cells was set as 100%. Upper panels: Western blot of HaCaT cells at 48 h post-transfection with the indicated TRAPPC8 siRNAs using anti-N1/603 and anti-α-tubulin antibody. (D) Effects of TRAPPC12 knockdown on gene transduction with 51PsVMaL2. HeLa cells were transfected with control, individual TRAPPC12 siRNAs (TTC15-01, -02, -03, or -04) or TRAPPC8 siRNA (KIAA1012-04). At 2 days after siRNA transfection the cells were inoculated with 51PsVMaL2 (MOI of ∼50 particles/cell). Following incubation for 2 days, the number of cells expressing GFP was measured by flow cytometry (left lower panel). Lysates prepared from HeLa cells at 48 h post-transfection were analyzed by Western blot using anti-TRAPPC12 antibody (left upper panel) or anti-TRAPPC8 antibody (anti-N1/603) (right panel). Alpha-tubulin was detected as a loading control. All experiments (A to D) were performed in triplicate. Error bars indicate standard deviations.

We examined the effect of TRAPPC8 knockdown on gene transduction with HPV51 PsV containing MaL1, MaL2, and the GFP-expression plasmid (51PsVMaL2). HeLa cells were individually transfected with four siRNAs against TRAPPC8 (KIAA1012-01, -02, -03, or -04), and two days later the TRAPPC8 levels were determined by Western blotting with an anti-TRAPPC8 antibody (anti-N1/603) purified from the serum of a rabbit immunized with recombinant TRAPPC8 N-terminal protein (aa 1–603, named N1/603). Expression of endogenous TRAPPC8 in cells transfected with TRAPPC8 siRNAs decreased at 48 h post-transfection compared to cells transfected with control siRNA (Figure 2A, upper panel). These TRAPPC8 knockdown cells were further inoculated with 51PsVMaL2, and 2 days later the number of cells expressing GFP was measured by flow cytometry. As shown in Figure 2A, the proportion of GFP-positive cells was reduced in TRAPPC8 knockdown cells compared to that in cells transfected with control siRNA. TRAPPC8 knockdown HeLa cells were viable 96 h post-transfection, (Figure 2A, right panel), indicating that the reduced GFP positivity was not due to impaired viability of the knockdown cells. As shown in Figure 2B, the number of GFP-positive cells also decreased in TRAPPC8 knockdown cells inoculated with HPV16 PsV (16PsV) and HPV31 PsV (31PsV).

We then tested the effect of TRAPPC8 knockdown on infection with authentic HPV virions. HaCaT cells were transfected with TRAPPC8 siRNAs (KIAA1012-03 or -04), and 2 days later the cells were inoculated with HPV31b virions prepared from CIN612 9E raft tissues. After an additional 3 day incubation, the levels of spliced viral E1ˆE4 transcripts were quantified by RT-qPCR. The levels of E1ˆE4 transcripts decreased in the TRAPPC8 knockdown cells compared to cells transfected with control siRNA, and this reduction correlated with the levels of TRAPPC8 knockdown (Figure 2C). These results suggest that TRAPPC8 is required for the early stages of native HPV infection.

We further examined the effect of TRAPPC12 knockdown on gene transduction with 51PsVMaL2. The proportion of GFP-positive cells was similarly reduced in TRAPPC12 knockdown cells (Figure 2D), implying the importance of TRAPPIII for HPV infection.

### Localization of TRAPPC8 on cell surface

**Figure 3:**
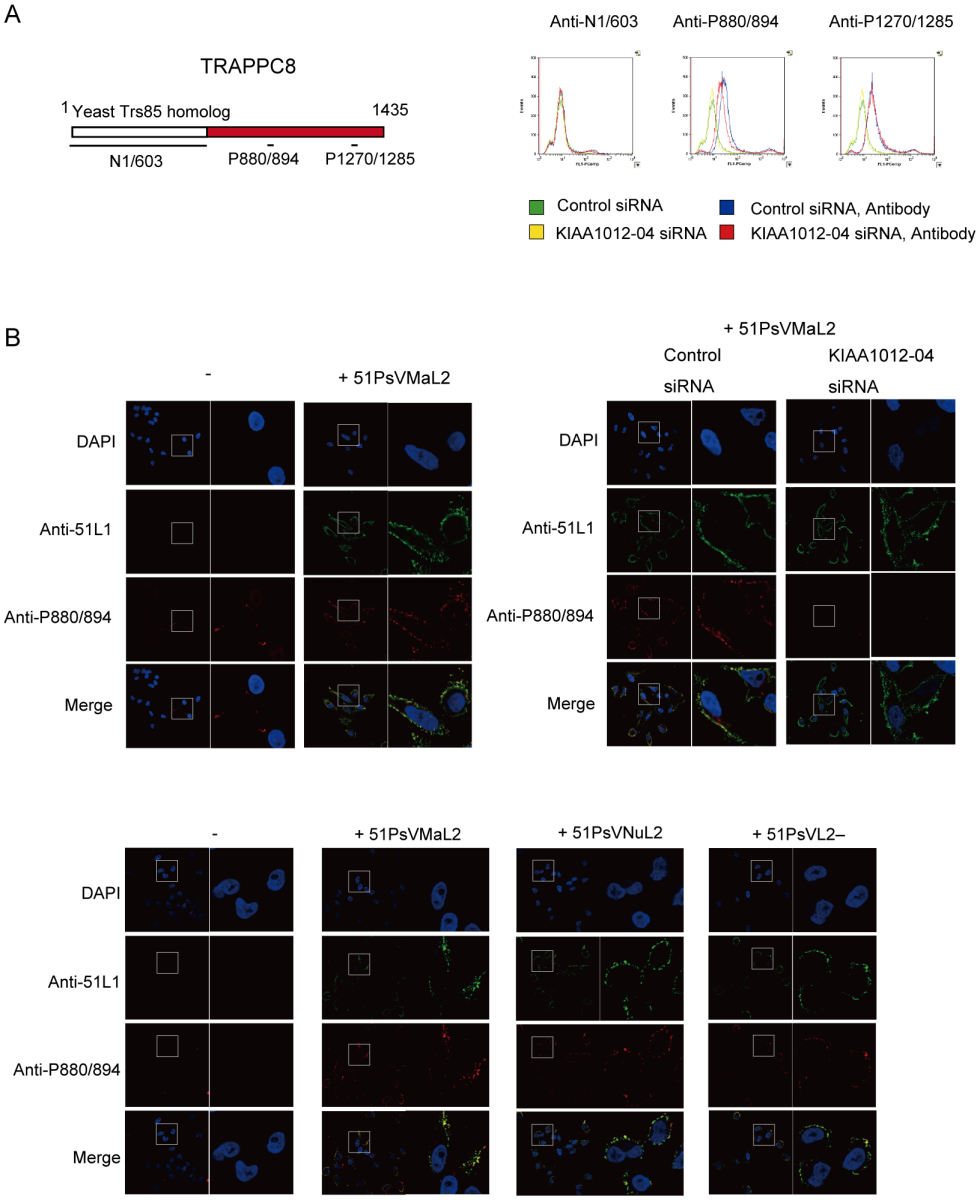
Localization of TRAPPC8 on the cell surface. (A) Flow cytometric analysis of cell-surface TRAPPC8. Schematic diagram of TRAPPC8 and the peptides used for rabbit immunization (upper panel). HeLa cells were transfected with control siRNA (green and blue lines) or TRAPPC8 siRNA (KIAA1012-04) (yellow and red lines). Two days later, cells were detached with EDTA in phosphate-buffered saline (PBS) and incubated with rabbit anti-N1/603, anti-P880/894, or anti-P1270/1285 (blue and red lines) in 10% fetal bovine serum in PBS at 4°C for 1 h. The cells were then incubated with Alexa Fluor 488-conjugated anti-rabbit IgG, and fluorescence was measured by flow cytometry (lower panels). (B) Confocal microscopy of cell-surface TRAPPC8. Left upper panel; HeLa cells were incubated in growth medium with or without 51PsVMaL2 (MOI of ∼2000 particles/cell) at 4°C for 1 h. Right upper panel; HeLa cells transfected with control or TRAPPC8 siRNA were inoculated with 51PsVMaL2 (MOI of ∼2000 particles/cell). Lower panel; HeLa cells were inoculated with or without 51PsVMaL2, 51PsVNuL2, or 51PsVL2– (MOI of ∼2000 particles/cell). These cells were then incubated in medium with mouse anti-51L1 antiserum and rabbit anti-P880/894 antibody, followed by staining with Alexa Fluor 488-conjugated anti-mouse IgG and Alexa Fluor 546-conjugated anti-rabbit IgG at 4°C. Cells were fixed, permeabilized, and mounted with Prolong Gold anti-fade reagent with DAPI. Fluorescence was examined using confocal microscopy. The boxed area is enlarged in the right panel.

To investigate potential roles for TRAPPC8 in HPV entry, we probed the plasma membrane for TRAPPC8 by using flow cytometry with three anti-TRAPPC8 antibodies: anti-N1/603, anti-P880/894, and anti-P1270/1285 (Figure 3A and S1A), which targets a TRAPPC8 peptide (aa 1270–1285, named P1270/1285; Figure 3A). HeLa cells were transfected with control siRNA (Figure 3A, green and blue lines) or TRAPPC8 siRNA (KIAA1012-04) (Figure 3A, yellow and red lines). Two days later, the cells were detached with EDTA and incubated with individual anti-TRAPPC8 antibodies (Figure 3A, blue and red lines) at 4°C for 1 h. The cells were then incubated with Alexa Fluor 488-conjugated anti-rabbit IgG, and fluorescence derived from the cells was analyzed by flow cytometry. As shown in Figure 3A, overall fluorescence intensity was increased in control siRNA-transfected cells probed with anti-P880/894 and anti-P1270/1285, and was slightly decreased in TRAPPC8 siRNA-transfected cells probed with anti-P880/894, but not with anti-P1270/1285. These results indicated that a proportion of TRAPPC8 is localized in the plasma membrane and that the epitope region of anti-P880/894 (aa 880–894) is exposed on the cell surface.

We further confirmed the localization of TRAPPC8 on the cell surface by confocal microscopy using anti-P880/894 (Figure 3B). HeLa cells were stained with anti-P880/894 and Alexa Fluor 546-conjugated anti-rabbit IgG; several areas of anti-P880/894 bound to the membrane surface were observed [Figure 3B, upper left panels (–)]. We then examined TRAPPC8 on the surface of cells inoculated with 51PsVMaL2. The cells were inoculated with 51PsVMaL2 at 4°C for 1 h, and stained with anti-P880/894 and anti-51L1 antiserum. A greater number of anti-P880/895 reactive locations were detected, which colocalized with anti-51L1 immunoreactivity (Figure 3B, upper left panels (+51PsVMaL2)). In contrast, no anti-P880/894 reactive locations were observed on the surface of cells transfected with TRAPPC8 siRNA (Figure 3B, upper right panels). Anti-N1/603 and anti-P1270/1285 immunoreactivity on the cell surface was barely detectable and did not colocalize with anti-51L1 immunoreactivity (Figure S1B), although anti-N1/603 immunoreactivity in permeabilized cells did colocalize with anti-51L1 and anti-P880/894 immunoreactivity (Figure S1C). Similar increases in anti-P880/894 immunoreactive locations, colocalized with anti-51L1 immunoreactivity, were observed on the surface of cells inoculated with 51PsVNuL2 or 51PsV lacking L2 (51PsVL2–) (Figure 3B, lower panels). These results suggest that the aa 880–894 region of TRAPPC8 is exposed on the cell surface in a manner that is L1 capsid inoculation-dependent, that it colocalizes with PsV and that this colocalization does not require interaction between L2 and TRAPPC8. Similar results were obtained with the commercially available anti-TRAPPC8 antibody, sc-8519 (Santa Cruz Biotechnology Inc.), which targets the C-terminal peptide of TRAPPC8 (Figure S1D); anti-TRAPPC8 immunoreactivity colocalized with L1 spots of on the cell surface when inoculated with 51PsVMaL2 (Figure S1E).

### Role of TRAPPC8 in PsV entry into cells

**Figure 4:**
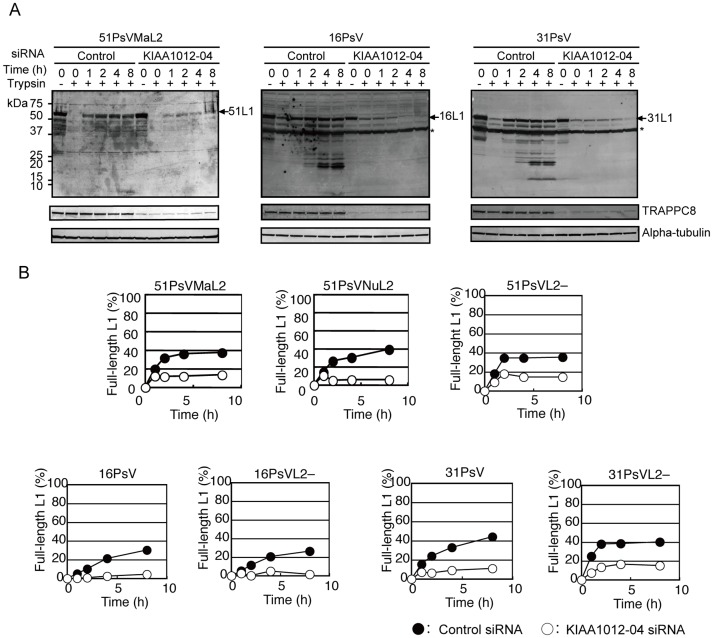
Effects of TRAPPC8 knockdown on PsV internalization. (A) HeLa cells transfected with control or TRAPPC8 siRNA (KIAA1012-04) were inoculated with PsV (MOI of ∼2000 particles/cell) and incubated for 1 h at 4°C. After washing with PBS, the cells were incubated in medium at 37°C for 0, 1, 2, 4, or 8 h. The cells were detached with PBS containing EDTA (trypsin –) or PBS containing trypsin and EDTA (trypsin +). The detached cells were lysed and boiled. HPV51 L1, TRAPPC8, and α-tubulin were detected by Western blotting using anti-51L1 antiserum, anti-TRAPPC8 (anti-N1/603), and anti-α-tubulin antibodies, respectively. HPV16 and HPV31 L1s were detected using anti-HPV16L1 antibody. Asterisks: unknown protein that reacted with the anti-HPV16L1 antibody. (B) Quantification of trypsin-resistant, full-length L1 in cells. HeLa cells transfected with control (black circle) or TRAPPC8 siRNA (KIAA1012-04) (white circle) were inoculated with 51PsVMaL2, 51PsVNuL2, 51PsVL2-, 16PsV, 16PsVL2–, 31PsV, or 31PsVL2– (MOI of ∼2000 particles/cell). After washing with PBS, the cells were incubated in the medium at 37°C for 0, 1, 2, 4, or 8 h. The cells were detached with PBS containing trypsin, and L1 in the cell lysates was detected by Western blotting (Figure S3B). Trypsin-resistant, full-length L1 was quantified using a Typhoon 9410 imager. The data for each PsV were obtained from one experiment.

Since our results indicated that TRAPPC8 knockdown severely impaired HPV infection and that TRAPPC8 colocalized with PsV on the cell surface following PsV inoculation, the potential role(s) of TRAPPC8 in PsV entry were investigated. Firstly, we examined the attachment of PsV in TRAPPC8 knockdown cells. HeLa cells transfected with TRAPPC8 siRNA (KIAA1012-04) were inoculated with 51PsVMaL2, 16PsV or 31PsV (Figure S2) and incubated for 1 h at 4°C. After removing unbound PsVs, the cells were detached with EDTA (Trypsin –, 0 h). PsVs bound to the cell surface were analyzed by Western blotting with anti-L1 antibodies. The L1 level was reduced by 0%–5% in cells transfected with TRAPPC8 siRNA compared to those transfected with control siRNA (Trypsin – at 0 h, Figures 4A and S3B). Although a decrease in L1 levels of 10%–30% was also observed in TRAPPC8 knockdown cells inoculated with HPV16 or HPV31 PsV (Trypsin – at 0 h, Figures 4A and S3B), these results suggest that TRAPPC8 does not play a major role in HPV cell attachment.

Next, we examined whether TRAPPC8 has a role in internalization of PsV. HeLa cells were incubated with 51PsVMaL2, 16PsV or 31PsV, in growth medium at 37°C for 0, 1, 2, 4, or 8 h. The cells were detached with trypsin, and L1 in the cell lysates analyzed by Western blotting with anti-L1 antibodies. As shown in Figure 4A, L1 was not detected in the lysates of cells harvested at time 0, indicating that PsV bound to the cell surface had not yet entered the cell. Following incubation with medium, trypsin-resistant L1 was detected, indicating that PsV had entered the cell (Figures 4A and S3B). In contrast, the level of trypsin-resistant L1 was severely reduced in cells transfected with TRAPPC8 siRNA (KIAA1012-04). This result was reproduced in cells transfected with another TRAPPC8 siRNA (KIAA1012-03) (Figure S3A). In addition, we performed the same experiments using 51PsVNuL2, 51PsVL2–, 16PsV lacking L2 (16PsVL2–), and 31PsV lacking L2 (31PsVL2–) (Figure S2). As shown in Figure 4B, all PsVs exhibited reduced levels of trypsin-resistant L1 in TRAPPC8 knockdown cells when compared with cells transfected with control siRNA. These results indicate that TRAPPC8 plays a crucial role in PsV internalization, but that the process is independent of L2.

**Figure 5:**
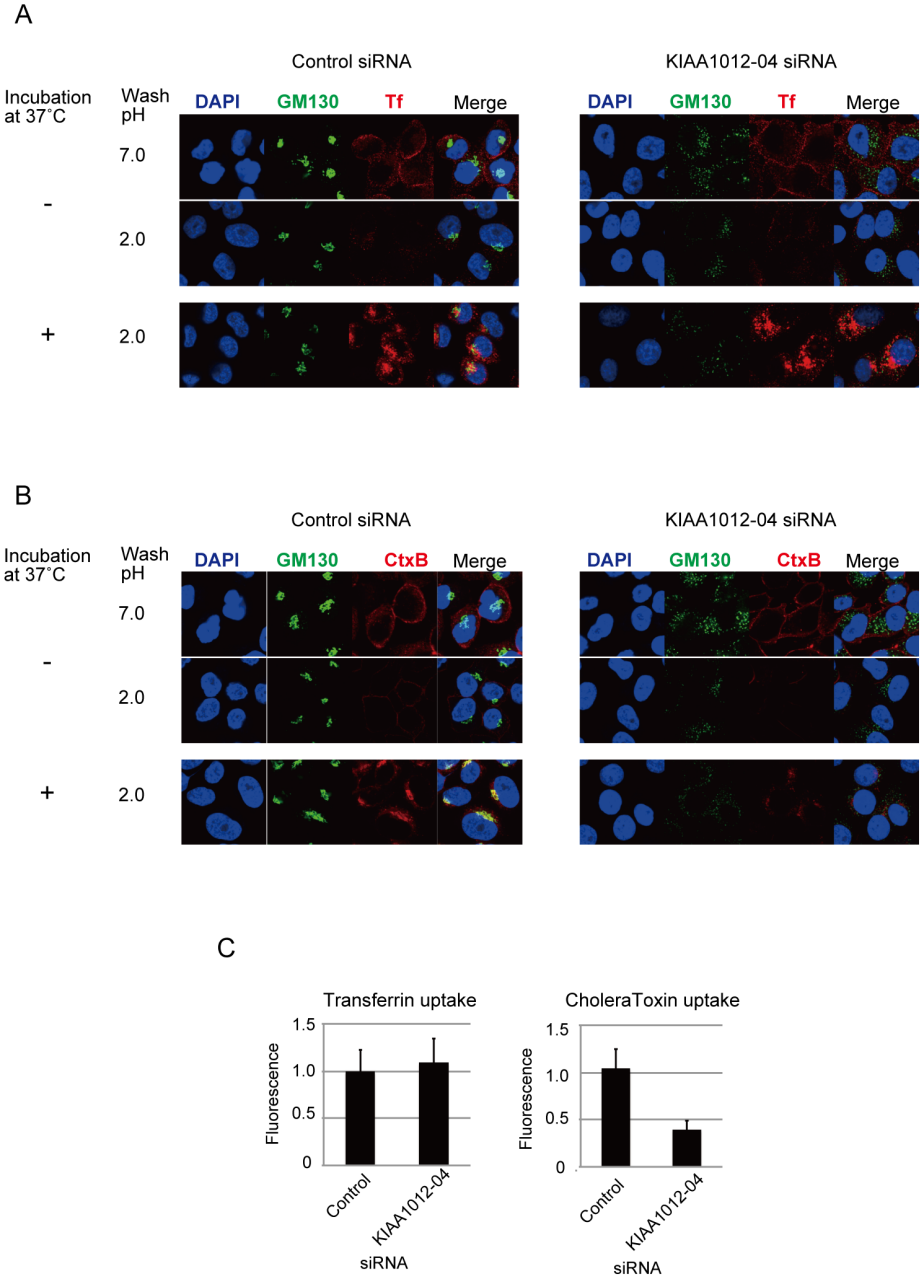
Effects of TRAPPC8 knockdown on uptake of transferrin and cholera toxin subunit B. (A, B) HeLa cells were transfected with control or TRAPPC8 siRNA (KIAA1012-04). The cells were incubated with transferrin (Tf) conjugated with Alexa Fluor 568 at 4°C for 1 h, and then incubated at 37°C for 15 min (A), or incubated with cholera toxin subunit B (CtxB) conjugated with Alexa Fluor 555 at 4°C for 1 h, and then incubated at 37°C for 1 h (B). Endocytotic uptake of the ligands was terminated by washing the cells with ice-cold PBS, and surface-bound Tf and CtxB were removed by acid treatment with DMEM containing 25 mM sodium acetate (pH 2.0) on ice for 2 min. The cells were fixed, permeabilized, and incubated with mouse anti-GM130 antibody (Golgi marker) and anti-mouse IgG conjugated with Alexa Fluor 488. (C) Quantification of uptake of Tf and CtxB. Fluorescence derived from incorporated Tf and CtxB was measured by confocal microscopy as the average fluorescence of three different fields (approximately 100 cells per field) from three independent experiments and quantified with FV1000 software (Olympus). The florescence intensity of incorporated Tf or CtxB obtained with control siRNA transfection was set 1. Error bars indicate standard deviations (n = 3).

We further examined whether TRAPPC8 knockdown causes general defects in the endocytic uptake of non-HPV molecules like transferrin (Tf) and cholera toxin subunit B (CtxB). While Tf is internalized through dynamin-dependent, clathrin-mediated endocytosis [53], CtxB is internalized via both dynamin-dependent, clathrin- or caveolae-mediated endocytosis and dynamin-independent endocytosis [53], [54]. TRAPPC8 knockdown in HeLa cells did not affect endocytic uptake of Tf (Figures 5A and 5C), suggesting that TRAPPC8 is not involved in dynamin-dependent, clathrin-mediated endocytosis. By contrast, transfection of TRAPPC8 siRNA caused partial defects in uptake of CtxB compared to control siRNA transfection (Figures 5B and 5C), implying that TRAPPC8 has a general role in either dynamin-independent or dynamin-dependent, caveolae-mediated endocytosis.

### Subcellular localization of L1 and packaged DNA in cells inoculated with HPV51 PsVs

**Figure 6:**
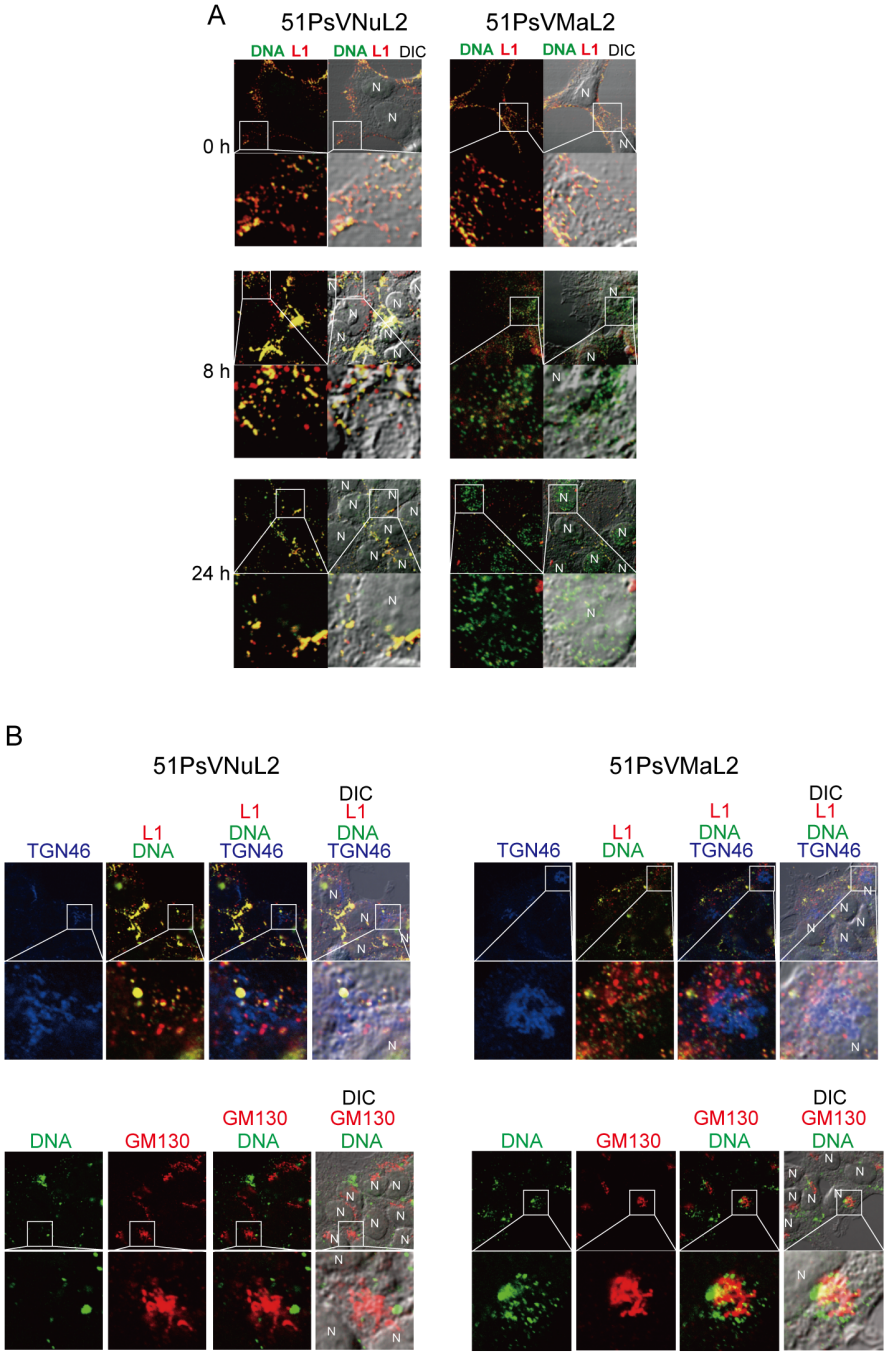
Subcellular localization of 51PsVs. (A) Subcellular localization of 51L1 and packaged DNA. HEK293FT cells were incubated with 51PsVNuL2 or 51PsVMaL2 (MOI of ∼2000 particles/cell) containing 5-ethynyl-2′-deoxyuridine (EdU)-labeled DNA in growth medium at 37°C for 0, 8, or 24 h. The cells were fixed and permeabilized. L1 was visualized with mouse anti-51Ll VLP antiserum and Alexa Fluor 555-conjugated anti-mouse IgG (red). EdU-labeled DNA was visualized with Alexa Fluor 488 azide (green). Fluorescent images were obtained using confocal and differential interference contrast (DIC) microscopy. The boxed area is enlarged below. Nucleus is shown as “N”. (B) Subcellular localization of EdU-labeled DNA, 51L1 and TGN46. HEK293FT cells were incubated with 51PsVNuL2 or 51PsVMaL2 in growth medium at 37°C for 8 h. The cells were fixed and permeabilized. EdU-labeled DNA was visulalized with Alexa Fluor 488 azide (green). L1 was visualized with mouse anti-51Ll VLP antiserum and Alexa Fluor 555-conjugated anti-mouse IgG (red). TGN46 (trans-Golgi marker) was visualized with rabbit anti-TGN46 antibody (ab50595, Abcam) and Alexa Fluor 647-conjugated anti-rabbit IgG (blue). In lower panels, instead of L1 staining, GM130 (Golgi marker) was visualized with mouse anti-GM130 antibody and Alexa Fluor 555-conhugated anti-rabbit IgG (red).

Because TRAPPC8 knockdown inhibited the internalization of PsVs in a manner independent of L2 interaction, we then investigated which stage of HPV infection requires L2-TRAPPC8 interaction. To this end, we monitored intracellular trafficking of 51PsVNuL2 or 51PsVMaL2 in the cell. HEK293FT cells were inoculated with these PsVs packaged with 5-ethynyl-2′-deoxyuridine (EdU)-labeled DNA. L1 and EdU-labeled DNA were visualized with the anti-51L1 antiserum and Click-it chemistry, respectively. In both 51PsVNuL2 and 51PsVMaL2-inoculated cells, EdU-labeled DNA and L1 colocalized (yellow dots, Figure 6A) on the cell surface at 0 h indicating cell attachment of the PsVs. After incubation for 8 h, EdU-labeled DNA (green dots, Figure 6A) was observed in the cytoplasm of 51PsVMaL2-inoculated cells, suggesting that PsV had been internalized and that its packaged DNA was separated from the L1 capsid (Figure 6A, right panel). In contrast, EdU-labeled DNA and L1 in the 51PsVNuL2-inoculated cells colocalized in the cytoplasm at 8 h. At 24 h, EdU-labeled DNA was transferred into the nucleus in 51PsVMaL2-inoculated cells, whereas EdU-labeled DNA and L1 in 51PsVMuL2-inoculated cells remained colocalized in the perinuclear region as large clusters without DNA translocation into the nucleus.

Since recent studies have revealed HPV trafficking to the trans-Golgi network (TGN) before genome translocation into the nucleus [33], [34], we examined whether localization of EdU-labeled DNA and L1 to the TGN differ between NuL2- and MaL2-containing PsVs. HEK293FT cells were inoculated with 51PsVNuL2 or 51PsVMaL2 and, after 8-h incubation, the TGN was visualized with anti-TGN46 antibody (trans-Golgi marker) together with staining of L1 and EdU-labled DNA. As shown in Figure 6B, while L1 staining was adjacent to or colocalized with the TGN similarly in both 51PsVNuL2 and 51PsVMaL2 (Figure 6B, upper panels, red dots), more EdU-labeled DNA signals were adjacent to the TGN in 51PsVMaL2-inoclated cells than in 51PsVNuL2-inoculated cells (Figure 6B, upper panels, green and yellow dots). Similar results were obtained by staining the cells with the Golgi marker, anti-GM130 antibody (Figure 6B, lower panels), suggesting that EdU-labeled DNA separated from L1 is more successfully localized in the Golgi in 51PsVMaL2-inoculated cells. These results raise the possibility that L2-TRAPPC8 interaction may be required for the viral genome localization in the TGN/Golgi compartment and that this L2 function could be related to the impaired infectivity observed in NuL2.

### Golgi dispersal caused by L2 expression and TRAPPC8 knockdown

**Figure 7:**
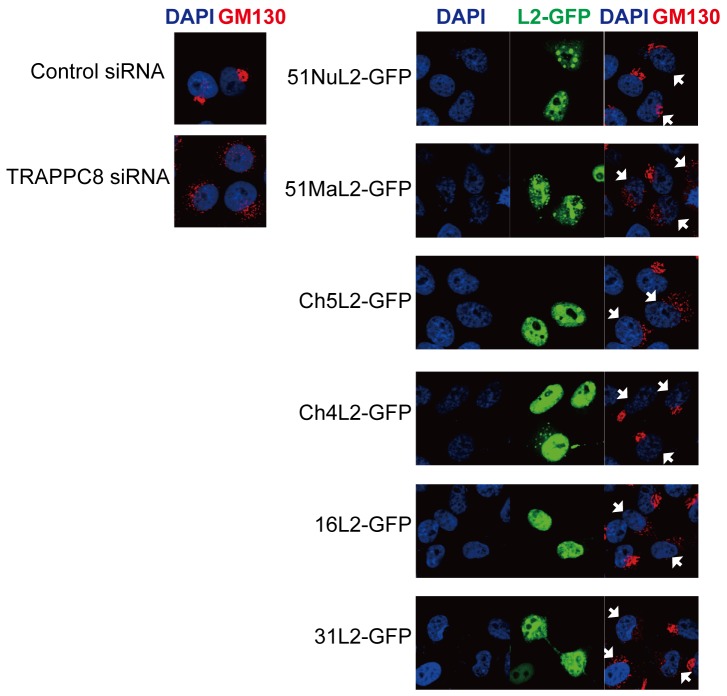
Effects of L2 expression on the Golgi structure. HeLa cells were transfected with control or TRAPPC8 siRNA (KIAA1012-04) (left panel), or expression plasmids for L2-GFP fusion proteins (right panel). After 24 h incubation, the cells were fixed and permeabilized, and stained with mouse anti-GM130 antibody (Golgi marker) and Alexa Fluor 555-conjugated anti-mouse IgG. Fluorescence in the cells was examined by confocal microscopy. The cells expressing L2-GFP are indicated by white arrows.

To further gain insight into roles for L2-TRAPPC8 interaction in the Golgi, we investigated Golgi morphology in cells expressing L2. HeLa cells were transfected with expression plasmids for a series of L2-GFP fusion proteins, and the Golgi bodies inside the cell visualized using anti-GM130 antibody. As shown in Figure 7, Golgi stacks were dispersed in the cells expressing MaL2-GFP, but not in the cells expressing NuL2-GFP. In contrast, immunofluorescence assays performed on cells expressing MaL2-GFP using the anti-early endosome antigen 1 (early endosome marker), anti-LAMP2 (late endosome marker) and anti-protein disulfide isomerase (ER marker) antibodies, did not reveal obvious structural changes in the corresponding organelles (Figure S4C). Golgi dispersal was also observed in cells expressing L2s that were capable of interacting with TRAPPC8, such as Ch5L2, HPV16 and HPV31 L2s (Figure 7, right panels). Intriguingly, TRAPPC8 knockdown in HeLa cells caused Golgi dispersal that was indistinguishable from that observed in MaL2-GFP expressing cells (Figure 7, left panels). Similarly, MaL2-GFP expression and TRAPPC8 knockdown did not affect early endosomes, late endosomes, or the ER (Figure S4A). Although we were unable to detect colocalization of PsV with TRAPPC8 in the Golgi compartment because of high anti-TRAPPC8 background staining of intracellular compartments (data not shown), these results suggest that the interaction between L2 and TRAPPC8 disrupts normal Golgi structure through inhibition of TRAPPC8 function.

## Discussion

In this study we have identified TRAPPC8, a specific subunit of the mammalian TRAPPIII complex (mTRAPPIII), as an L2-interacting protein, and demonstrated that the knockdown of TRAPPC8 in human epithelial cells reduces both gene transduction with PsV and infectivity of authentic HPV virions. These observations strongly suggest that TRAPPC8 plays a central role in the early stages of HPV infection. However, TRAPPC8 knockdown inhibited the internalization of PsV independently of L2, thus excluding the requirement for L2-TRAPPC8 interaction during the initial entry step. Further, we find that the L2-TRAPPC8 interaction can induce Golgi dispersal and that this Golgi phenotype strongly correlates with the gene transduction efficiency of PsV. Thus, we propose two non-overlapping, L2-independent and -dependent roles for TRAPPC8 in the HPV cell entry mechanism.

**Figure 8:**
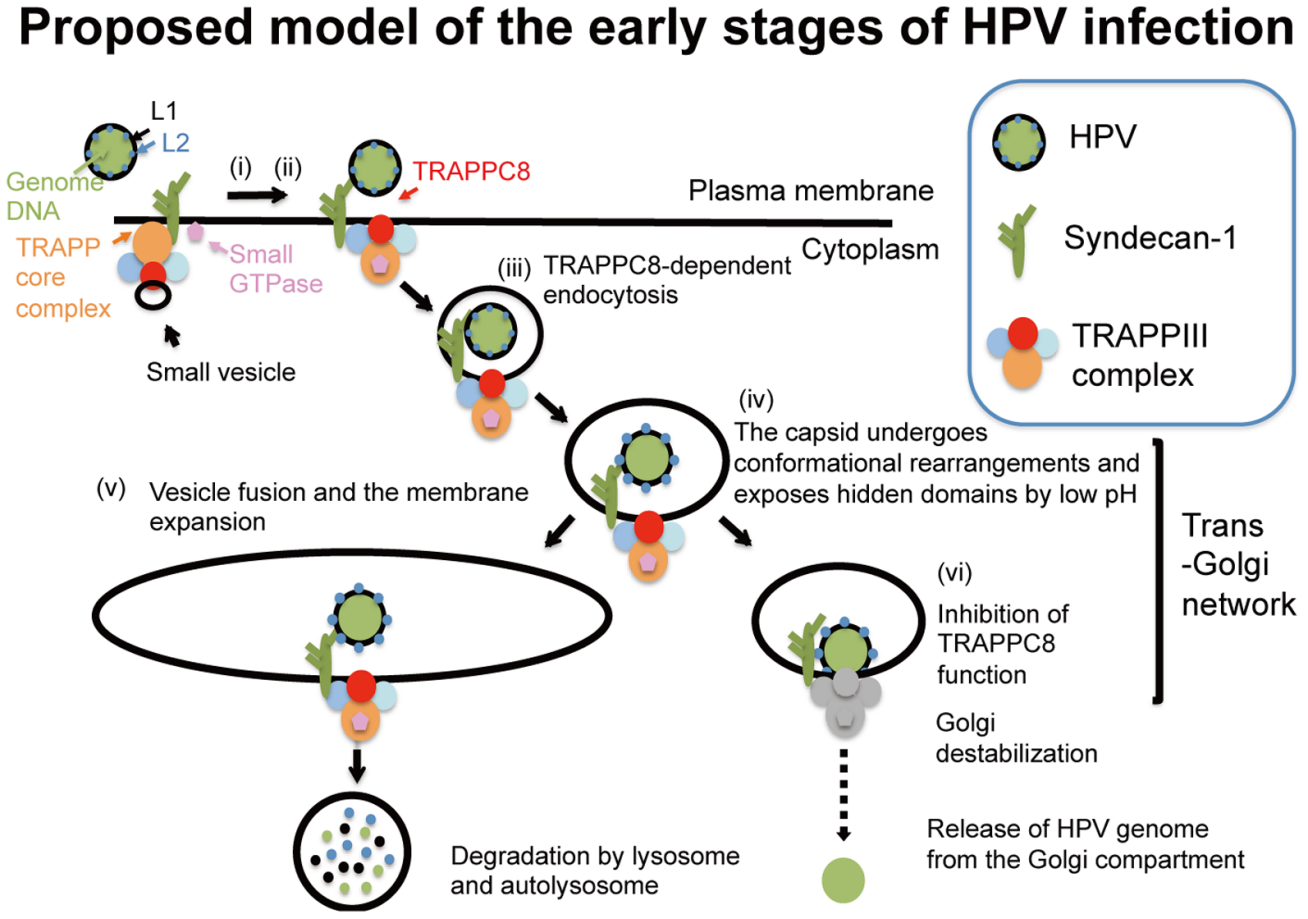
Proposed model for TRAPPC8 involvement in HPV infection. (i) HPV binds to HSPGs on the epithelial cell surface. (ii) The central region of TRAPPC8 is exposed on the cell surface upon virion attachment. (iii) The capsid is internalized into the cell by TRAPPC8-dependent endocytosis. (iv) The capsid undergoes conformational rearrangements and exposes hidden domains when exposed to low pH, leading to an exposure of the L2 N-terminal region. (v) Endosomal vesicles containing HPV51 Nu or HPV that fails to interact with TRAPPC8 are elongated or fused to each other, and these virions are eventually degraded by lysosomes and autolysosomes. (vi) HPV that interacts with TRAPPC8 through L2 inhibits TRAPPC8 functions, such as vesicle fusion and membrane expansion, both of which are necessary for the subsequent degradation process, thereby leading to the release of the viral genome from the trans-Golgi network.

For the L2-independent role of TRAPPC8, we propose a model in which the HPV virion, trapped by cell-surface L1-binding proteins such as HSPGs, is internalized by TRAPPC8-dependent endocytosis (Figure 8). The central region of TRAPPC8 was exposed on the surface of HeLa cells and colocalized with inoculated PsV (Figure 3B). These observations suggest the possibility that TRAPPC8 is located adjacent to HSPGs on the cell surface. Syndecans (syndecan-1 to -4) are a major class of membrane-spanning HSPGs, and their transmembrane (TM) and C-terminal cytosolic domains are highly conserved. Among the syndecans, syndecan-1 acts as an initial attachment protein for HPV entry into keratinocytes [55]. Given that TRAPPC4, one of the TRAPP core subunits, binds to the conserved EFYA motif at the C-terminus of syndecan-2 [56], it is possible that mTRAPPIII containing TRAPPC4 also binds to syndecan-1 in association with virion. Furthermore, TRAPPC8-containing vesicles beneath the plasma membrane may fuse with the plasma membrane trapping HPV, increasing exposure of its central region on the cell surface (Figure 3B). When considering the mechanism of TRAPPC8-dependent endocytosis, it is worth noting that TRAPP complexes regulate the function of small-GTPases via their GEF activity and by the combination of individual TRAPP subunits [43]. Thus, mTRAPPIII may regulate the GTPase activity of Rab5 and/or other unknown small GTPases that are necessary for the endocytosis of HPV [19], [32].

TRAPPC8 knockdown did not affect uptake of transferrin (Figure 5), excluding the possibility that the effects of TRAPPC8 knockdown are non-specific and act on multiple endocytotic pathways. However, TRAPPC8 knockdown partially decreased uptake of cholera toxin, which is internalized via both dynamin-dependent and -independent endocytosis (Figure 5). Since TRAPPC8 knockdown reduces the cell's susceptibility to ricin [45], which enters the cell via a dynamin-independent pathway [54], and HPV also enters the host keratinocyte via a dynamin-independent pathway [19], TRAPPC8 may have a general role in this endocytotic pathway.

TRAPP complexes are involved in both the transport of vesicles from the ER to the Golgi and the endosome to the Golgi [43], and mTRAPPIII plays important roles in Golgi stack formation [47]. Recently, Day *et al.* and Lipovsky *et al.* reported that HPV16 is delivered to the TGN or the Golgi during the entry process [33], [34], highlighting the importance of the TGN/Golgi compartment as a route for infectious HPV entry. Intriguingly, the Golgi dispersal phenotype observed in cells expressing L2s able to bind TRAPPC8 was almost identical to that seen in TRAPPC8 knockdown cells (Figures 7 and S4), suggesting that the interaction of L2 with TRAPPC8 inhibits its function in the Golgi. In contrast, L2s that can interact with TRAPPC8 did not affect early endosomes, late endosomes, or the ER (Figure S4C), which suggests a specific effect of L2-TRAPPC8 interaction on the Golgi structure. In summary, we speculate that the L2-TRAPPC8 interaction inhibits TRAPPC8 function in the Golgi and causes Golgi destabilization, thereby facilitating escape of the HPV genome from the Golgi compartment.

Since most regions in L2 molecules are buried in the capsid and the N-terminus of L2 becomes exposed on the capsid surface during the entry process [1], [11], the L2 N-terminus including region I may interact with TRAPPC8 in the lumen of endocytotic vesicles. Although we were unable to detect direct interactions between recombinant TRAPPC8 and L2 (data not shown), TRAPPC12, another specific subunit contained in mTRAPPIII, showed similar L2-binding profiles to TRAPPC8 (Figure 1C, right panel), suggesting that mTRAPPIII binds to L2 as an entire complex. Further support for this mechanism comes from the observation that the knockdown of TRAPPC12 in HeLa cells reduced susceptibility to gene transduction with 51MaL2PsV (Figure 2D). Similar results were obtained in HeLa cells transfected with siRNA against TRAPPC11, another subunit in mTRAPPIII (data not shown). These results suggest that mTRAPPIII as a whole plays an important role in HPV infection. In contrast, HeLa cells transfected with siRNA against TRAPPC9, one of the mTRAPPII subunits, exerted little or no effect on gene transduction with PsV (data not shown), suggesting that mTRAPPII is not involved in HPV infection. Moreover, the effects of siRNAs for each TRAPP subunit (TRAPPC8, 9, 11, and 12) on HPV infection show similar susceptibility to ricin, suggesting that HPV and ricin share the same entry pathway, a pathway that is dependent on mTRAPPIII.

Human TRAPPC8 has been shown to contribute to the autophagy system [46] and its yeast homolog, Trs85, is required for the formation of pre-autophagosomal structure and the membrane expansion of autophagosomes [43], [44], [57]. Thus the L2-TRAPPC8 interaction may lead to the arrests of vesicle fusion or membrane expansion at the TGN. These arrests are likely to protect HPV trapped in the lumen of the vesicle from subsequent degradation by lysosomes or autophagosomes. This model is further supported by recent findings that autophagy responses are induced in the initial stages of HPV16 infection [58], and that most internalized HPVs are degraded by autolysosomes [59], [60]. We hypothesize that while most HPVs are degraded by the cell defense machinery, including the autophagy system, a proportion of internalized HPVs, i.e. those in which L2 binds to TRAPPC8, inhibit its function and thus escape from the TGN through Golgi destabilization (see Figure 8). NuL2, which lacks the capacity to bind to TRAPPC8, may not be able to avoid such degradation. Although further experiments are needed to substantiate our hypothetical models for the roles of TRAPPC8 in infectious HPV entry, our findings will contribute to a better understanding of the mechanisms by which HPV enters host cells and escapes from the TGN.

## Supporting Information

Figure S1 **Immunofluorescence microscopy analysis for TRAPPC8 on the cell surface.** (A) Western blotting of lysates prepared from HeLa cells transfected with control or TRAPPC8 siRNA (KIAA1012-04) using anti-TRAPPC8 antibodies (anti-N1/603, anti-P880/894, and anti-P1270/1285). Upper panel: schematic diagram of TRAPPC8 and the peptides used for rabbit immunization. (B) HeLa cells were incubated with 51PsVMaL2 (MOI of ∼2000 particles/cell) in growth medium at 4°C for 1 h. After washing with medium, the cells were incubated in medium with mouse anti-51L1 VLP antiserum and rabbit anti-N1/603 (left panel) or anti-P1270/1285 (right panel), followed by staining with Alexa Fluor 488-conjugated anti-mouse IgG and Alexa Fluor 546-conjugated anti-rabbit IgG. The cells were fixed and permeabilized, then mounted with Prolong Gold anti-fade reagent with DAPI. (C) HeLa cells were incubated with 51PsVMaL2 (MOI of ∼2000 particles/cell) in growth medium at 4°C for 1 h. After removing unbound PsVs, the cells were incubated in medium with mouse anti-51L1 VLP antiserum and rabbit anit-P880/894, followed by staining with Alexa Fluor 488-conjugated anti-mouse IgG and Alexa Fluor 546-conjugated anti-rabbit IgG. The cells were fixed and permeabilized, then incubated with or without rabbit anti-N1/603, followed by staining with Alexa Fluor 647-conjugated goat anti-rabbit IgG. Fluorescence was visualized by confocal microscopy. The boxed areas are enlarged in the right panels. (D) Western blot analysis using commercial anti-TRAPPC8 antibody, sc-85191 (Santa Cruz Biotechnology Inc.). Truncated TRAPPC8 proteins, aa 1–603 (N1/603), aa 604–1435 (C604/1434), aa 604–747 (P604/747), aa 737–886 (P737/886), aa 876–1025 (P876/1025), aa 1015–1164 (P1015/1164), aa 1154–1303 (P1154/1303), and aa 1293–1435 (P1293/1435), were expressed in *E.coli* Rosetta-gami B (Takara Bio Inc.) by using the pCold II vector system (Takara Bio Inc.) and purified by nickel affinity chromatography. These proteins were electrophoresed and stained with CBB (upper panel). The proteins were analyzed by Western blotting using sc-85191 (lower panel). (E) Immunofluorescence microscopy analysis for cell-surface TRAPPC8 using sc-85191. HeLa cells were incubated with 51PsVMaL2 (MOI of ∼2000 particles/cell) in growth medium at 4°C for 1 h. After removing unbound PsVs, the cells were incubated in medium with mouse anti-51L1 VLP antiserum and goat anti-TRAPPC8 antibody, sc-85191, followed by staining with Alexa Fluor 488-conjugated anti-mouse IgG and Alexa Fluor 546-conjugated anti-goat IgG. The cells were fixed and permeabilized, then incubated with rabbit anti-N1/603, followed by staining with Alexa Fluor 647-conjugated goat anti-rabbit IgG. Fluorescence was visualized by confocal microscopy. The boxed areas are enlarged in the right panels.
(TIF)

Figure S2 **Characterization of PsVs.** (A) Electrophoresis analysis of PsV fractions prepared from HEK293FT using the Opti-Prep gradient method as described in Materials and Methods. Proteins in the PsV fractions were stained with SYPRO Ruby. The arrows indicate the protein bands corresponding to L1 or L2. Right panel: molecule ratio between L1 and L2 in PsV fractions. (B) Electron micrograph of PsVs. The PsV fractions were settled on carbon-coated copper grids negatively stained with 2% uranyl acetate. The grids were examined using a Hitachi model H-7650 transmission electron microscope. (C) Ratio of DNase-resistant reporter plasmid to total reporter plasmid packaged in PsVs. PsV fractions were incubated with DNase-I, and DNase-resistant DNA was quantified by qPCR with the following primers complementary to the reporter plasmid pEF1α-EGFP: 5'-GCG GCC GCG CCA CCA TGG TGA GCA AGG GCG AGG AGC-3' and 5'-AAG CTT ACT TGT ACA GCT CGT CCA TGC CGA G-3'.
(TIF)

Figure S3 **Effects of TRAPPC8 knockdown on PsV internalization.** (A, B) HeLa cells transfected with control or TRAPPC8 siRNAs (KIAA1012-03 or -04) were inoculated with 51PsVMaL2, 51PsVNuL2, 51PsVL2–, 16PsV, 16PsVL2–, 31PsV, or 31PsVL2– (MOI of ∼2000 particles/cell) and incubated for 1 h at 4°C. After washing with PBS, the cells were incubated in medium at 37°C for additional 0, 1, 2, 4 or 8 h. The cells were detached with PBS containing EDTA (Trypsin –) or PBS containing trypsin and EDTA (Trypsin +) at the indicated time points. The detached cells were lysed and boiled. Type 51L1, 16L1, 31L1, TRAPPC8, or α-tubulin were detected by Western blotting using anti-51MaL1 VLP antiserum, anti-HPV16L1 antibody (554171; BD Biosciences), anti-TRAPPC8 (anti-N1/603) and anti-α-tubulin antibodies, respectively. Asterisks: unknown protein that reacted with the anti-HPV16L1 antibody. Alpha-tubulin was detected as a loading control.
(TIF)

Figure S4 **Effects of TRAPPC8 knockdown or 51MaL2 expression on intracellular organelles.** (A) Effects of TRAPPC8 knockdown on early endosomes, late endosomes, or the endoplasmic reticulum (ER). HeLa cells transfected with control or TRAPPC8 siRNA (KIAA1012-04) were incubated in medium at 37°C for 2 days. The cells were fixed, permeabilized, and incubated with anti-EEA1 (early endosome marker, 610457; BD Biosciences), anti-LAMP2 (late endosome marker, 555803; BD Biosciences) or anti-PDI (ER marker, ab2729; Abcam) antibody, followed by staining with Alexa Fluor 555-conjugated anti-mouse IgG, and mounted with Prolong Gold with DAPI. Fluorescence in the cells was examined by confocal microscopy. (B, C) Effects of expression of 51MaL2-GFP on early endosomes, late endosomes, or the ER. HeLa cells transfected with pCMV-GFP (B) or pCMV-51MaL2-GFP (C) were incubated in medium at 37°C for 24 h. The cells were fixed, permeabilized, and incubated with anti-GM130 (Golgi marker, 610822; BD Biosciences), anti-EEA1, anti-LAMP2, or anti-PDI antibody, followed by staining with Alexa Fluor 555-conjugated anti-mouse IgG, and mounted as described above. Fluorescence in the cells was examined by confocal microscopy. White arrows indicate cells expressing GFP or 51MaL2-GFP.
(TIF)
