# Supplementary figures and images for: Identification of TRAPPC8 as a Host Factor Required for Human Papillomavirus Cell Entry
Source: PLoS One. 2013 Nov 14;8(11):e80297. doi: 10.1371/journal.pone.0080297 (PMC3828182; doi:10.1371/journal.pone.0080297)

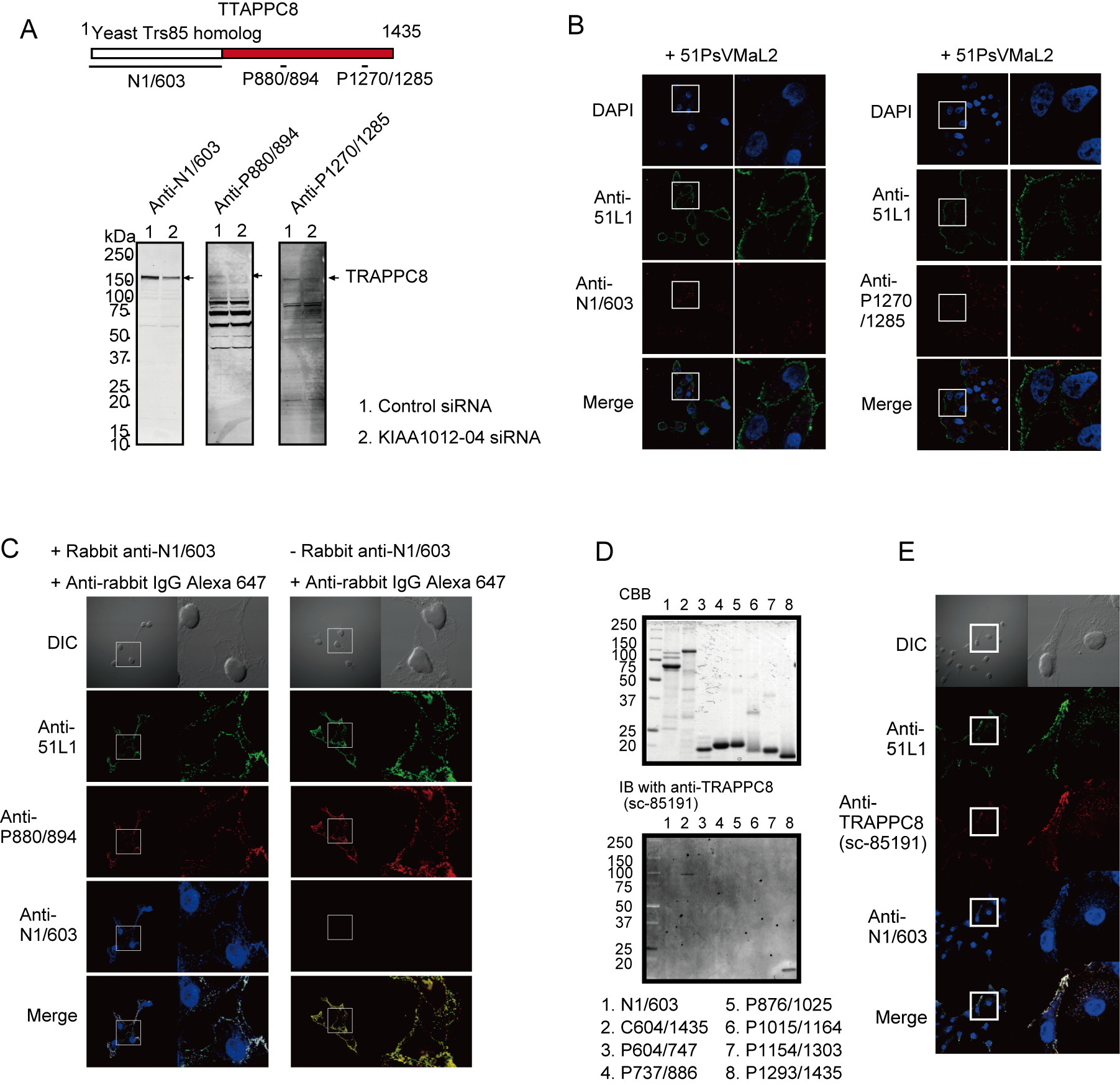

Supplement: Figure S1 — Immunofluorescence microscopy analysis for TRAPPC8 on the cell surface. (A) Western blotting of lysates prepared from HeLa cells transfected with control or TRAPPC8 siRNA (KIAA1012-04) using anti-TRAPPC8 antibodies (anti-N1/603, anti-P880/894, and anti-P1270/1285). Upper panel: schematic diagram of TRAPPC8 and the peptides used for rabbit immunization. (B) HeLa cells were incubated with 51PsVMaL2 (MOI of ∼2000 particles/cell) in growth medium at 4°C for 1 h. After washing with medium, the cells were incubated in medium with mouse anti-51L1 VLP antiserum and rabbit anti-N1/603 (left panel) or anti-P1270/1285 (right panel), followed by staining with Alexa Fluor 488-conjugated anti-mouse IgG and Alexa Fluor 546-conjugated anti-rabbit IgG. The cells were fixed and permeabilized, then mounted with Prolong Gold anti-fade reagent with DAPI. (C) HeLa cells were incubated with 51PsVMaL2 (MOI of ∼2000 particles/cell) in growth medium at 4°C for 1 h. After removing unbound PsVs, the cells were incubated in medium with mouse anti-51L1 VLP antiserum and rabbit anit-P880/894, followed by staining with Alexa Fluor 488-conjugated anti-mouse IgG and Alexa Fluor 546-conjugated anti-rabbit IgG. The cells were fixed and permeabilized, then incubated with or without rabbit anti-N1/603, followed by staining with Alexa Fluor 647-conjugated goat anti-rabbit IgG. Fluorescence was visualized by confocal microscopy. The boxed areas are enlarged in the right panels. (D) Western blot analysis using commercial anti-TRAPPC8 antibody, sc-85191 (Santa Cruz Biotechnology Inc.). Truncated TRAPPC8 proteins, aa 1–603 (N1/603), aa 604–1435 (C604/1434), aa 604–747 (P604/747), aa 737–886 (P737/886), aa 876–1025 (P876/1025), aa 1015–1164 (P1015/1164), aa 1154–1303 (P1154/1303), and aa 1293–1435 (P1293/1435), were expressed in E.coli Rosetta-gami B (Takara Bio Inc.) by using the pCold II vector system (Takara Bio Inc.) and purified by nickel affinity chromatography. These proteins were electrophoresed and s [file pone.0080297.s001.tif]

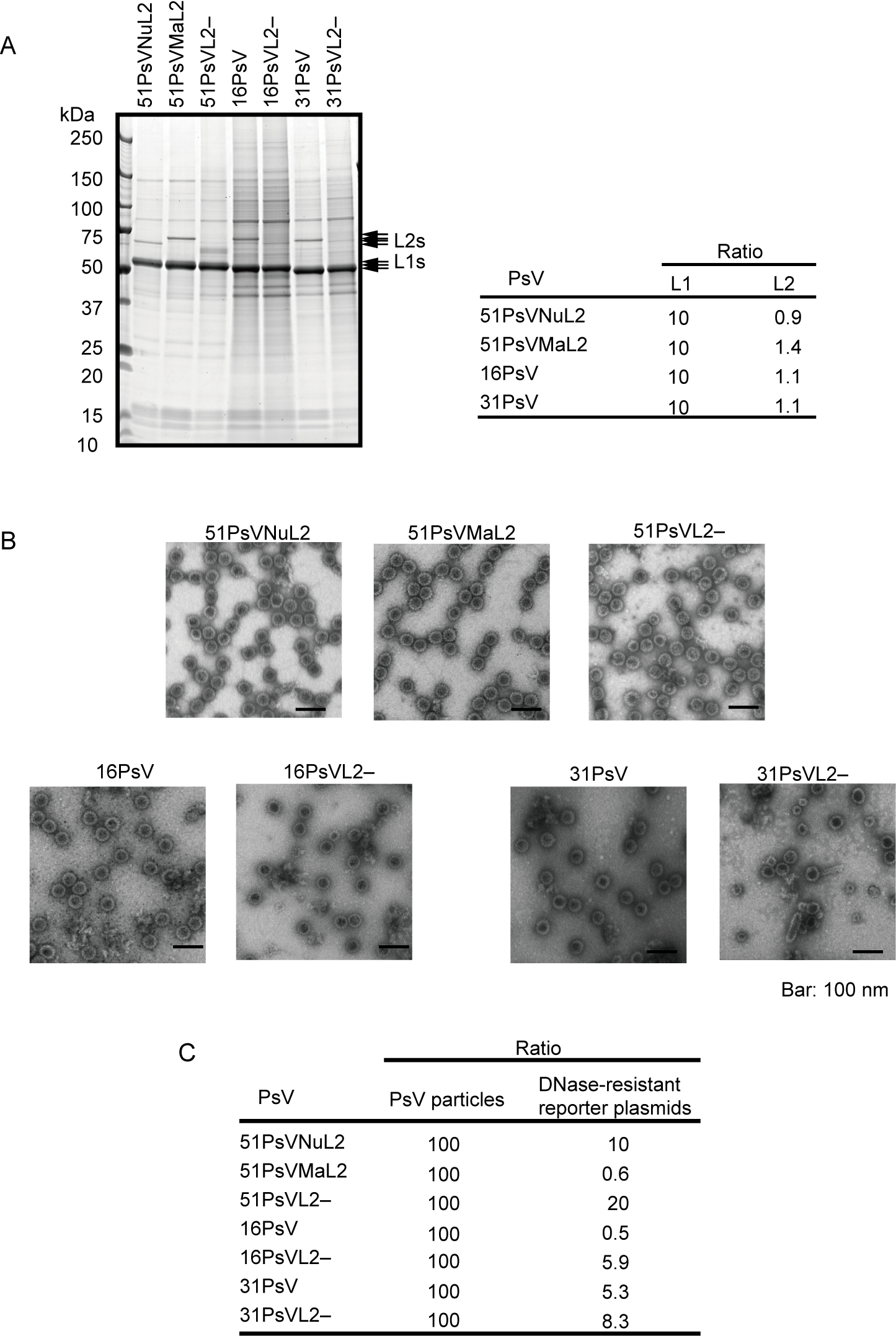

Supplement: Figure S2 — Characterization of PsVs. (A) Electrophoresis analysis of PsV fractions prepared from HEK293FT using the Opti-Prep gradient method as described in Materials and Methods. Proteins in the PsV fractions were stained with SYPRO Ruby. The arrows indicate the protein bands corresponding to L1 or L2. Right panel: molecule ratio between L1 and L2 in PsV fractions. (B) Electron micrograph of PsVs. The PsV fractions were settled on carbon-coated copper grids negatively stained with 2% uranyl acetate. The grids were examined using a Hitachi model H-7650 transmission electron microscope. (C) Ratio of DNase-resistant reporter plasmid to total reporter plasmid packaged in PsVs. PsV fractions were incubated with DNase-I, and DNase-resistant DNA was quantified by qPCR with the following primers complementary to the reporter plasmid pEF1α-EGFP: 5'-GCG GCC GCG CCA CCA TGG TGA GCA AGG GCG AGG AGC-3' and 5'-AAG CTT ACT TGT ACA GCT CGT CCA TGC CGA G-3'. (TIF) [file pone.0080297.s002.tif]

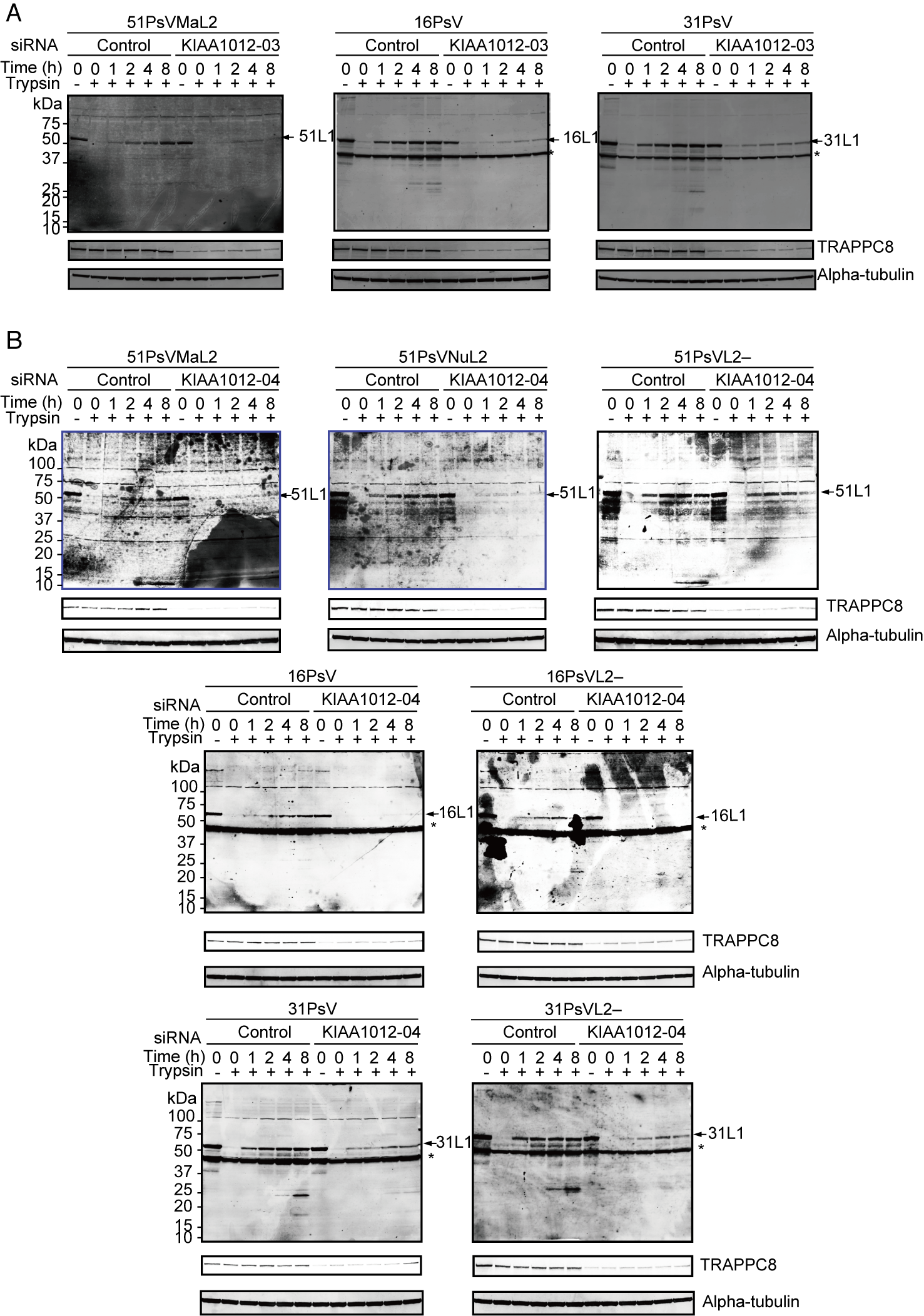

Supplement: Figure S3 — Effects of TRAPPC8 knockdown on PsV internalization. (A, B) HeLa cells transfected with control or TRAPPC8 siRNAs (KIAA1012-03 or -04) were inoculated with 51PsVMaL2, 51PsVNuL2, 51PsVL2–, 16PsV, 16PsVL2–, 31PsV, or 31PsVL2– (MOI of ∼2000 particles/cell) and incubated for 1 h at 4°C. After washing with PBS, the cells were incubated in medium at 37°C for additional 0, 1, 2, 4 or 8 h. The cells were detached with PBS containing EDTA (Trypsin –) or PBS containing trypsin and EDTA (Trypsin +) at the indicated time points. The detached cells were lysed and boiled. Type 51L1, 16L1, 31L1, TRAPPC8, or α-tubulin were detected by Western blotting using anti-51MaL1 VLP antiserum, anti-HPV16L1 antibody (554171; BD Biosciences), anti-TRAPPC8 (anti-N1/603) and anti-α-tubulin antibodies, respectively. Asterisks: unknown protein that reacted with the anti-HPV16L1 antibody. Alpha-tubulin was detected as a loading control. (TIF) [file pone.0080297.s003.tif]

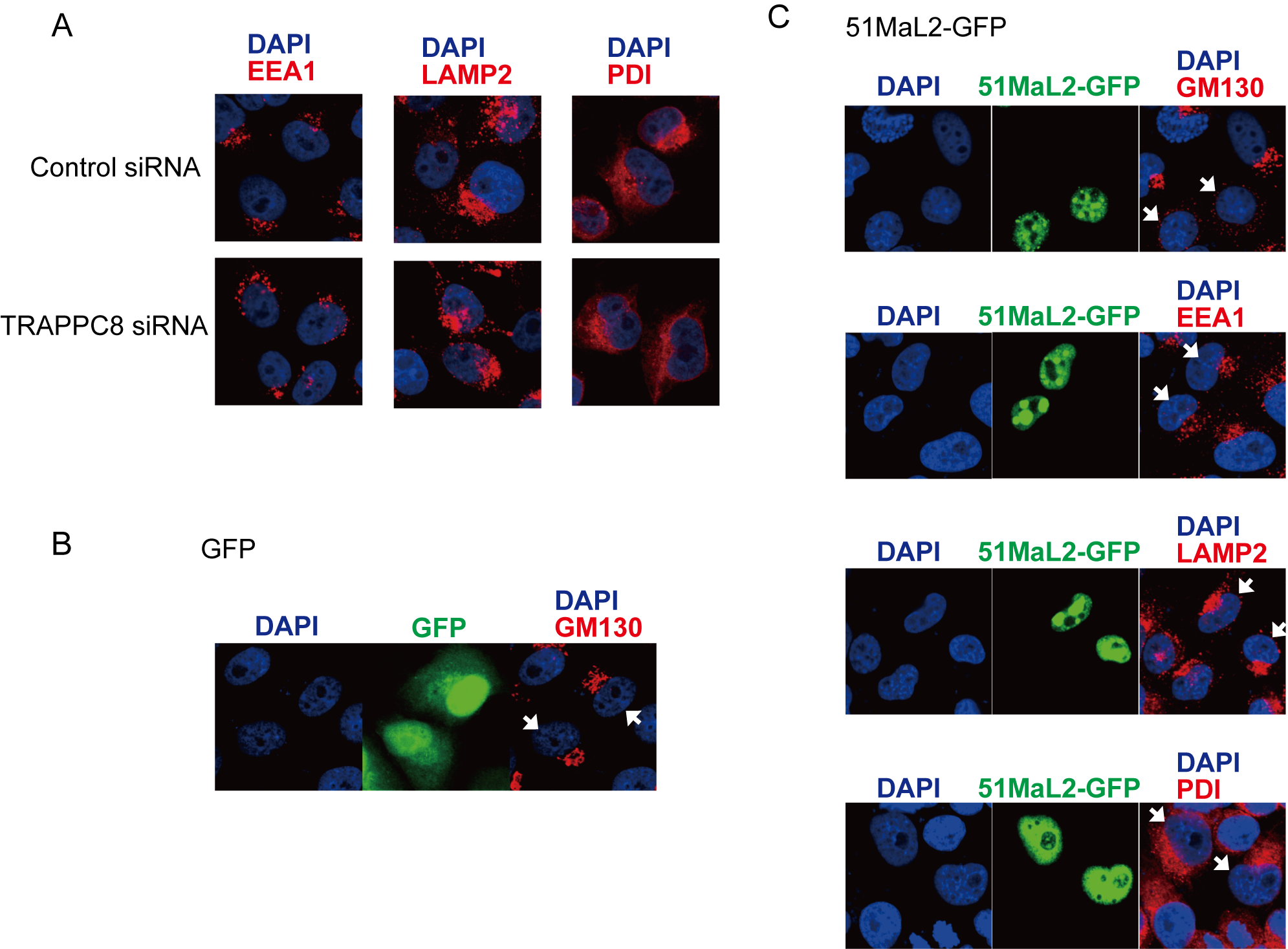

Supplement: Figure S4 — Effects of TRAPPC8 knockdown or 51MaL2 expression on intracellular organelles. (A) Effects of TRAPPC8 knockdown on early endosomes, late endosomes, or the endoplasmic reticulum (ER). HeLa cells transfected with control or TRAPPC8 siRNA (KIAA1012-04) were incubated in medium at 37°C for 2 days. The cells were fixed, permeabilized, and incubated with anti-EEA1 (early endosome marker, 610457; BD Biosciences), anti-LAMP2 (late endosome marker, 555803; BD Biosciences) or anti-PDI (ER marker, ab2729; Abcam) antibody, followed by staining with Alexa Fluor 555-conjugated anti-mouse IgG, and mounted with Prolong Gold with DAPI. Fluorescence in the cells was examined by confocal microscopy. (B, C) Effects of expression of 51MaL2-GFP on early endosomes, late endosomes, or the ER. HeLa cells transfected with pCMV-GFP (B) or pCMV-51MaL2-GFP (C) were incubated in medium at 37°C for 24 h. The cells were fixed, permeabilized, and incubated with anti-GM130 (Golgi marker, 610822; BD Biosciences), anti-EEA1, anti-LAMP2, or anti-PDI antibody, followed by staining with Alexa Fluor 555-conjugated anti-mouse IgG, and mounted as described above. Fluorescence in the cells was examined by confocal microscopy. White arrows indicate cells expressing GFP or 51MaL2-GFP. (TIF) [file pone.0080297.s004.tif]
